# Recent Trends in Advanced Radiation Shielding Concrete for Construction of Facilities: Materials and Properties

**DOI:** 10.3390/polym14142830

**Published:** 2022-07-12

**Authors:** Muhd Afiq Hizami Abdullah, Raizal Saifulnaz Muhammad Rashid, Mugahed Amran, Farzad Hejazii, N. M. Azreen, Roman Fediuk, Yen Lei Voo, Nikolai Ivanovich Vatin, Mohd Idzat Idris

**Affiliations:** 1 Department of Civil Engineering, Faculty of Engineering, Universiti Putra Malaysia, Serdang 43400, Selangor, Malaysia; raizal@upm.edu.my (R.S.M.R.); farzad@upm.edu.my (F.H.); 2 Department of Civil Engineering Technology, Faculty of Civil Engineering, Technology, University Malaysia Perlis, Perlis 02100, Malaysia; 3 Department of Civil Engineering, College of Engineering, Prince Sattam Bin Abdulaziz University, Alkharj 11942, Saudi Arabia; 4 Department of Civil Engineering, Faculty of Engineering and IT, Amran University, Quhal 9677, Yemen; 5 Material and Structural Integrity Group, Malaysia Nuclear Agency, Kajang 43600, Selangor, Malaysia; noorazreen@nuclearmalaysia.gov.my; 6 Polytechnic Institute, Far Eastern Federal University, 690922 Vladivostok, Russia; fedyuk.rs@dvfu.ru; 7 Peter the Great St. Petersburg Polytechnic University, 195251 St. Petersburg, Russia; vatin@mail.ru; 8 DURA Technologies Sdn. Bhd, Jalan Chepor 11/8, Pusat Seramik Fasa 2, Ulu Chepor, Chemor 31200, Perak, Malaysia; vooyenlei@dura.com.my; 9 School of Applied Physics, Faculty of Science and Technology, National University of Malaysia, Selangor 43600, Malaysia; idzat@ukm.edu.my

**Keywords:** additives, gamma ray shielding, heavy aggregates, neutron shielding, radiation shielding concrete

## Abstract

Nuclear energy offers a wide range of applications, which include power generation, X-ray imaging, and non-destructive tests, in many economic sectors. However, such applications come with the risk of harmful radiation, thereby requiring shielding to prevent harmful effects on the surrounding environment and users. Concrete has long been used as part of structures in nuclear power plants, X-ray imaging rooms, and radioactive storage. The direction of recent research is headed toward concrete’s ability in attenuating harmful energy radiated from nuclear sources through various alterations to its composition. Radiation shielding concrete (RSC) is a composite-based concrete that was developed in the last few years with heavy natural aggregates such as magnetite or barites. RSC is deemed a superior alternative to many types of traditional normal concrete in terms of shielding against the harmful radiation, and being economical and moldable. Given the merits of RSCs, this article presents a comprehensive review on the subject, considering the classifications, alternative materials, design additives, and type of heavy aggregates used. This literature review also provides critical reviews on RSC performance in terms of radiation shielding characteristics, mechanical strength, and durability. In addition, this work extensively reviews the trends of development research toward a broad understanding of the application possibilities of RSC as an advanced concrete product for producing a robust and green concrete composite for the construction of radiation shielding facilities as a better solution for protection from sources of radiation. Furthermore, this critical review provides a view of the progress made on RSCs and proposes avenues for future research on this hotspot research topic.

## 1. Introduction

In recent times, nuclear energy has come from the splitting of atoms through a fission or fusion reaction or through the decaying process. This energy can be used to generate steam that powers turbines, which have become a promising alternative to hydrocarbon or fossil fuel power-generating plants [1]. The health care, agriculture, and infrastructure utility sectors have also largely benefited from this energy source in terms of X-ray imaging and non-destructive tests [2]. However, nuclear energy, which is produced from a nuclear reaction, emits nuclear radiation. Nuclear radiation is defined as all elementary particles, either charged or uncharged, that possess energy in excess of 100 eV [3]. These particles include elementary particles that have acquired energy in ways other than through a nuclear reaction, such as electrostatic accelerators. Radiation can penetrate certain objects and has a harmful effect on any living organism. Nuclear radiation could cause skin burns, a reduction in white blood cells, and cell destruction, which leads to cancer [4]. Skin burns and nausea are classified as deterministic effects that are due to cell destruction and a slow cell division rate. Cancer is a stochastic effect that is due to cell modifications that result in malignancies [5]. This effect is hereditary and statistically detected in mammals exposed to radiation. Radiation eventually leads to the destruction of the ecosystem and renders a certain area inhabitable. One of the measures in protecting against radiation exposure is to provide proper shielding from nuclear radiation. Conventionally, lead is used as a shielding material. However, because lead itself is hazardous, a strong justification has been presented for opting for better alternative materials [6]. 

Concrete has been used in the construction of nuclear power plants since before 1975 [7]. It has certain advantages, such as low permeability to protect embedded steel reinforcement, durability against fire, ubiquitous materials for forming concrete, and flexibility to form any molded shapes. Concrete is mostly constructed as a protection dome in the primary containment, which contains the ionizing facility, and also in the secondary containment, which houses the turbine [8]. It is also used to construct dry cask storage, which stores spent nuclear fuel to prevent nuclear waste contamination [9]. Concrete is also used to construct trenches and shielding boxes of “hot cells” for managing radioactive waste in India [10]. This type of concrete is classified as radiation shielding concrete (RSC). RSC, also known as atomic energy protection concrete or heavyweight concrete (concrete made with magnetite aggregates can have a density of 3.2–4 t/m^3^, which is much higher than that of concrete made with ordinary aggregates), is a cement-based composite prepared with water, cement, and heavy weight aggregates [11]. The main purpose of the RSC design is to shield against neutrons and gamma rays.

**Figure 1:**
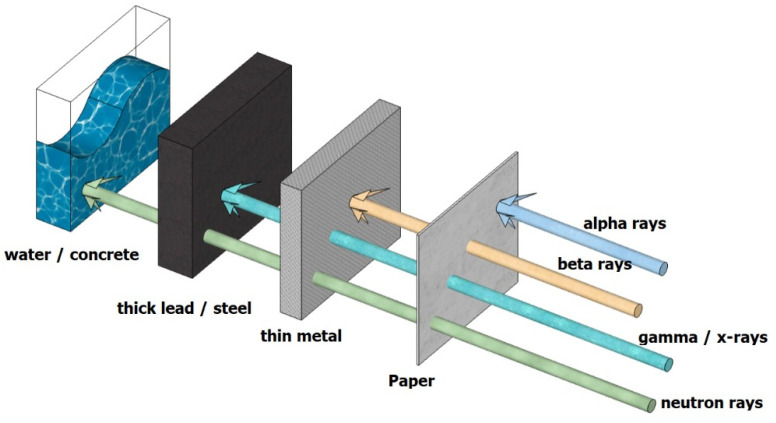
Type of radiations, penetrations, and their properties (Adapted with improvements from [21]).

The direction of recent research has focused on concrete’s ability to attenuate harmful energy radiating from nuclear sources through various alterations to its composition [12,13,14,15,16]. Nuclear sources radiate four kinds of radiation: alpha particles, beta particles, gamma rays, and neutrons (Figure 1) [12,17,18]. Alpha particles consist of two protons and two neutrons, which are emitted by naturally occurring heavyweight elements such as uranium or radium [19]. Beta particles can be either electrons or positrons and are emitted by many radioactive elements [20]. These two types of radiation do not cause a body or any material to become radioactive as they can be blocked by a sheet of paper or a few millimeters of wood [21]. Gamma rays and neutrons are more penetrative and are widely discussed in studies on RSC [22,23]. Most previous scholars collected data on concrete mixtures of varying ranges and studied various concrete mixtures used as radiation shields by using heavyweight aggregates of different minerals to find the linear attenuation coefficient (*µ*) experimentally and theoretically [24]. 

According to researchers, concrete with magnetite fine aggregates has higher physicomechanical characteristics than concrete with barite and goethite [17]. Changes in cement aggregates have an impact on the structure and radiation shielding efficacy of concrete. Several studies have examined the manufacture of high-density concretes that can provide enhanced radiation shielding while preserving a modest thickness [25,26,27,28,29].

This aim can be achieved by employing particular additives rather than modifying the aggregate concentration. Concretes with additives in the aggregate can shield against rays more effectively than plain concrete can [30]. In 2015, researchers examined various shielding properties, including five ores: barite, serpentine, magnetite limonite, and hematite. Barite and magnetite were determined to be suitable for X-ray shielding [28]. Researchers often choose one type of aggregate to be obtained experimentally, such as ilmenite [31], barite [26,32,33,34,35,36], lime/silica [37], hematite [38], zeolite [38], lead glass [39], lead mine waste [27], and magnetite [17,40]. Different photon energies produce different amounts of energy; many experiments are required to acquire only one for each photon energy. When several minerals are compared, e.g. fly ash, silica fume, rice husk ash, etc., it is computed theoretically; this theoretical computation has been conducted by using simulation tools such as XCOM [25,29,41], MicroShield [30], and a Monte Carlo simulation [36].

Recent researchers have also investigated the viability of developing ultra-high-performance concrete (UHPC) with radiation shielding properties. UHPC is deemed one of the most sophisticated concrete technologies today and is a cementitious composite with compressive and flexural strengths that exceed 200 and 20 MPa, respectively [18,24,42,43,44,45,46,47,48]. The flexural tensile strength of UHPC is reported to exceed 21 MPa [49]. Hot-pressed cement [50] was the first high-performance cement, followed by macro-defect-free cement [51], slurry-infiltrated fiber concrete [52], and related materials. Afterward, researchers introduced reactive powder concrete (RPC), the compression strength of which increased from 200 MPa to 800 MPa by improving fineness and reactivity [22]. RPC is expensive and is, therefore, not widely used in the industry. Larrard [26] proposed UHPC in 1994, which has a high compressive strength because of its high density. The good mechanical properties of UHPC are achieved by reducing the water-to-cement ratio to less than 0.2, by using the aggregate with a fine particle size, and by high temperature curing [49]. This approach leads to fewer internal pores and the compact microstructure of UHPC. Research on UHPC as an RSC or ultra-high-performance RSC (UHPRSC) reported a strength reduction due to the replacement of silica sand with a fine-sized heavyweight aggregate [24]. Furthermore, the reported gamma attenuation value was much lower than that of the high-strength concrete at the same energy exposure and of the same heavyweight aggregate type [18,53]. Experimental studies on the neutron shielding of UHPRSC and the shielding property at elevated temperatures are lacking. These parameters are vital in assessing the viability of UHPRSC in ionizing facilities such as nuclear power plants. Given the merits of RSCs, this article presents a comprehensive review on the subject, considering the classifications, alternative materials, additives, and type of heavy aggregates used. These parameters defined the mechanical strength, shielding properties, and durability of RSC. Hence, this literature review provides critical reviews on the RSC performance in terms of radiation shielding characteristics, strength, and durability properties. In addition, development research trends toward a broad understanding of the application possibilities of RSC as an advanced concrete product for producing a robust and green concrete composite for the construction of radiation shielding facilities were extensively reviewed.

## 2. Classifications and Major Functions of RSC

RSC is a composite prepared with water, cement, and heavyweight aggregates [18,24]. With its high amount of crystallized water and high density, it can shield against radiations such as beta rays, alpha rays, gamma rays, neutrons, and X-rays [39]. Beta and alpha rays have the lowest penetrating capabilities of these photons [54]. Thinner shielding materials can quickly absorb photons. Therefore, the primary goal of the RSC design is to protect against gamma rays and neutrons. Gamma and X-rays are electromagnetic waves with a high penetrating capability. They can be absorbed by weighty materials or dense concrete [55]. Thus, heavy elements, namely, elements with a large atomic weight, are required in RSC [56,57]. Neutrons are atoms with no electric charge, a high amount of energy, and the ability to penetrate deeply [13]. Neutrons are divided into three types based on their energy: low neutrons, moderate neutrons, and fast neutrons. Fast neutrons can decelerate and can even be protected after colliding with heavy atoms [11]. Light elements, such as boron and hydrogen, can absorb low neutrons and intermediate neutrons. Therefore, not only heavy materials, but also light elements are required in neutron RSC [58]. Concrete (e.g., RSC) is perhaps the most frequently used radiation shielding material because it can be molded easily into complicated shapes and is more appropriate for neutron and proton shielding than other shielding materials [31]. 

**Figure 2:**
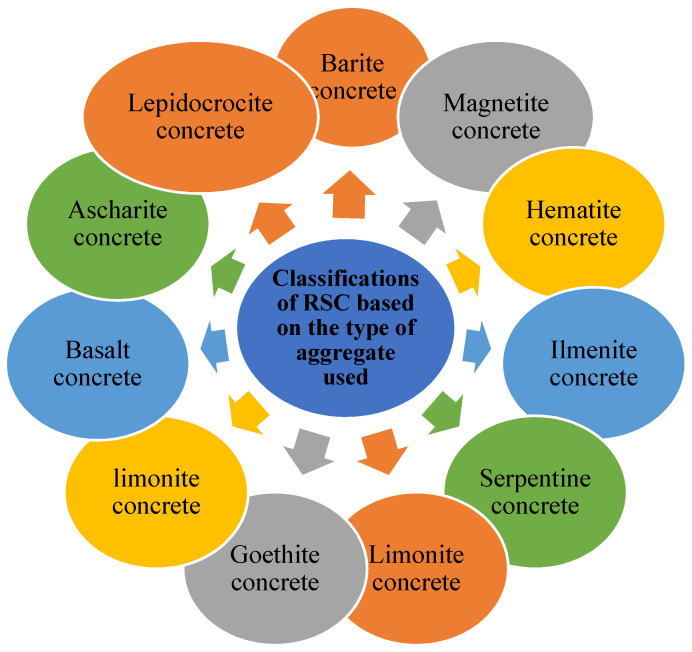
Classifications of RSC (Adapted with improvements from [21]).

Radiation shielding provides three main functions: (1) thermal shielding to protect the coolant loop, pressure vessel, and inner shield from the intense heat produced by nuclear radiation absorption, (2) biological shielding to ensure public health and personnel protection, and (3) instrument and apparatus shielding to protect electronic instruments and electromagnetic apparatus [59]. RSC is mostly used in the construction of facilities related to nuclear radiation, including nuclear power plants, spent nuclear fuel storage, and radiation imagery and therapy facility. RSC is widely used to construct such facilities because most materials that constitute RSC are vastly available. In nuclear power generating facilities, RSC has been used for containment structures lined with steel surrounding a reactor or steam generator [8]. RSC that is used to protect a reactor needs to have a high content of heavyweight coarse aggregate to attenuate radiation [7]. For the secondary containment, which includes the steam generator, prestressed concrete is required to contain the high pressure generated from the pressurized steam generator [8]. Figure 2 shows the classifications of RSC.

RSC is also used in the storage of spent nuclear fuel in nuclear power plants. Once the spent nuclear fuel is removed from the reactor, it is stored in the water of a concrete pool lined with stainless steel to reduce the temperature [8]. Spent fuel can also be stored in dry cask storage, which provides more mobility for the waste and has no risk of water leakage [60]. Another type of nuclear waste storage is reinforced concrete trenches with a wall thickness that varies from 350–750 mm. This type of storage requires waterproofing to prevent the ingress of groundwater. 

In health facilities, such as hospitals, structures are mostly made of reinforced concrete. Hence, a section that houses a radiation facility, such as a proton therapy room, is constructed by using concrete that can also be categorized as RSC [61]. The RSC wall is equipped with borated-polyethylene shields and a steel door to prevent radiation leakage [62]. RSC is commonly used for nuclear- and radiation-related facilities because of the widely available low-cost materials that constitute the RSC.

## 3. Alternative Materials Used in RSC

**Figure 3:**
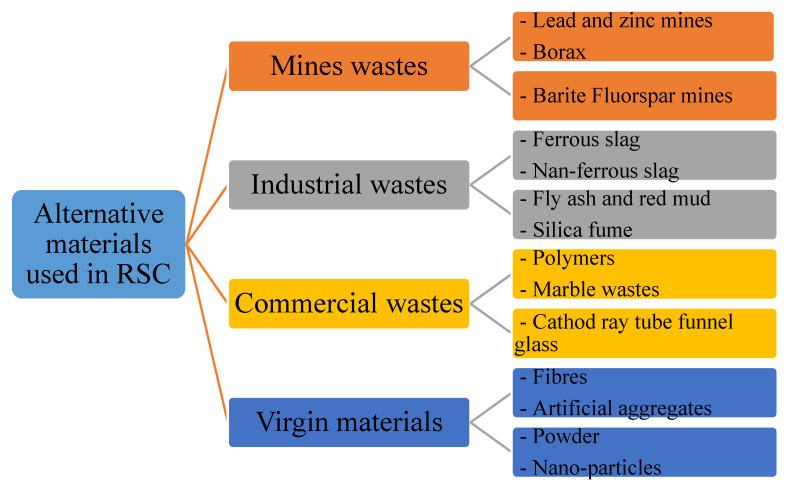
Alternative materials used in RSC (adapted with improvements from [21]).

The composition of RSC is mostly similar to that of conventional concrete except for its aggregates. Certain RSCs incorporate a heavyweight aggregate to improve their radiation shielding property, hence requiring less thickness for a containment structure [7]. Similar to conventional concrete, RSC is made of a binder, aggregate, and additive. The alternative materials used by RSC researchers can be classified based on their origins, which include industrial wastes, mine wastes, commercial wastes, and alternate virgin materials (Figure 3). Alternate materials are also classified based on their usage patterns, such as whether they are used as a substitute for cement or aggregate.

### 3.1. Binder

As in conventional concrete, a binder serves as a binding agent between aggregates. The binder that is commonly used in concrete is cement. Dry cement powder reacts with water to form the binder, and the mix ratio between the cement and water defines the water-to-cement ratio, which influences the concrete strength. The amount of cement used in research ranges from 269–1423 kg per cubic meter (kg/m^3^) of concrete produced [63,64]. The amount of cement used can affect the matrix of concrete, hence influencing the mechanical properties of the concrete. Partial cement replacement material such as fly ash and silica fume is also studied in RSC. Research on RSC with a cathode-ray tube as the aggregate used 62 kg/m^3^ of fly ash, which constituted 14.9% of the total binder content [65]. Another study on RSC with recycled concrete as the aggregate used 798 kg/m^3^ of fly ash, which is 64% of the total binder content [66]. The study also used ground granulated blast furnace slag as the binder, the amount varying from 179–283 kg/m^3^, which is 14–21% of the total binder content. Silica fume is also included in the mix but is very minimal at 35 kg/m^3^. Another study on RSC with varied aggregates used 50 kg/m^3^ of silica fume, which is 10% of the overall binder content [67]. A study on UHPRSC used a large volume of silica fume in the mixture at 200 kg/m^3^, which is 19.5% of the total binder content.

### 3.2. Aggregate

The aggregate used for RSC has various types and sizes. These properties influence the mechanical strength and shielding properties of RSC. The aggregate used in RSC can be categorized as a lightweight natural aggregate, heavyweight natural aggregate, natural aggregate with crystallized water, slag, mines, and industrial waste. Table 1 shows the composition, performance, and density of the heavyweight aggregates.

#### 3.2.1. Natural Aggregate/Low-Density Aggregate

Lightweight natural aggregate can be considered a low-density aggregate and is commonly used in concrete. Such aggregate includes granite, limestone, sandstone, silica sand, and dolomite. This type of aggregate has mostly light elements among its components, such as calcium and silicon [67,72,73]. The density of this aggregate ranges from 2600–2760 kg/m^3^ [67,72,74,75]. A high percentage of light elements in the composition and the low density of this type of aggregate resulted in a lower density of RSC produced, thus reducing the shielding properties.

#### 3.2.2. Natural Heavyweight Aggregate

Heavyweight aggregate or high-density aggregate is material with a density exceeding 3000 kg/m^3^, according to BS 8110 and EN 206-1 [76]. BS 6110 is a high-density aggregate, which is aggregate with a bulk density of more than 4000 kg/m^3^. In developing a high-density concrete, a heavyweight aggregate provides a major advantage over a lightweight naturally occurring aggregate. Naturally occurring heavyweight aggregates include barite, magnetite, hematite, geothite, limonite, and ilmonite.

Barite is often found in veins associated with lead and zinc mineral ore deposits that contain mostly BaSO_4_ [76]. This type of aggregate has a relative density between 4.0–4.3; the 90% purity of this aggregate produced concrete with a minimum density of 3400 kg/m^3^ [17,76,77]. However, a high amount of barite fines in a concrete mixture may delay the setting of the concrete. The high density of barite is contributed by the high amount of BaSO_4_, which contains the heavy element of barium, as represented by the percentage of purity.

For example, magnetite contains iron oxide (Fe_3_O_4_) at a percentage between 49.6–90%, which is linked to a density between 2860–4800 kg/m^3^ [53,76,78]. The purities of magnetite indicated by the high percentage of Fe_3_O_4_ define its density, which is higher than that of barites.

Another type of heavyweight aggregate is ilmenite ore, which contains iron and titanium. At a percentage of 35.55–65.74% and 21–23.08% for Fe_2_O_3_ and TiO_2_, respectively, ilmenite has a density of 4200–4240 kg/m^3^ [76,79,80]. Hematite is also common in RSC research, but it is often reported to have a lower density than that of barite and magnetite. This type of aggregate has a high percentage of Fe_2_O_3_ between 27–81.13%, thereby indicating a density range between 2900–2967 kg/m^3^ [18,76,81].

Another naturally occurring low-density aggregate is goethite, with a bulk density of 2100 skg/m^3^ and a relative density of 3.5 [76]. Goethite contains iron oxide (hydrated, FeO[OH]) with 63% of iron. Limonite also has a lower density than that of barite and magnetite, with a bulk density of 2200 kg/m^3^ and a relative density of 3.7 [76]. Ilmenite also contains iron oxide (hydrated), 2Fe_2_O_3_.3H_2_O, with 60% of iron.

#### 3.2.3. Natural Aggregate with Crystallized Water

Other specialized aggregates that are being used in research related to RSC and nuclear industries are colemanite and serpentine. These aggregates are mostly related to the shielding of neutron radiation because of the presence of hydrogen and boron in these ores, which is effective in slowing down and capturing neutron particles [77]. Serpentine contains crystallized water in its structure and is very stable even at high temperatures compared to goethite and limonite [76]. Colemanite has a monoclinic crystal structure with a chemical formula of 2CaO.3B_2_O_3_.5H_2_O, and it contains hydrogen and boron [82]. Colemanite is the main source in the production of borax and boric acid. 

#### 3.2.4. Synthetic Aggregate

A synthetic aggregate is produced for the purpose of increasing the shielding property of produced RSC. This increase is achieved by producing a heavyweight aggregate or aggregate that contains neutron-absorbing elements. An iron shot is a type of synthetic aggregate made from chilled iron or steel and can be customized to have a certain density or maximum value [76]. A steel shot has a density of 7850 kg/m^3^. A study used up to 40% of the total aggregate with a steel shot that has a density of 2300–4200 kg/m^3^, while another study produced RSC with 100% aggregate composed of a steel shot with a density of 3480–3680 kg/m^3^ [83,84]. Ferro boron is another type of synthetic aggregate that is being researched to improve the attenuation of RSC. It has been studied using powder and a coarse-sized aggregate [85]. Ferro boron is a binary alloy of iron and boron produced through the carbothermic reduction of boric acid and low-carbon steel. The heavy element of iron attenuates fast neutrons, while the presence of boron, which has a high-absorption cross section, can capture the neutrons. Ferro boron contains 10–20% boron with 72.2% of iron [85,86].

#### 3.2.5. Mine Wastes

Mine wastes are also investigated as an aggregate in RSC due to their density and presence of heavy elements. Barite-fluorspar mine waste (BFMW) is a type of waste generated from the mining industry and contains trace elements of lead, zinc, and cadmium [87]. The major element in BFMW is CaO, at 25.74%, followed by SiO_2_, Al_2_O_3_, and Fe_2_O_3_ at 14.33%, 2.84%, and 2.5%, respectively. The density of BFMW is 3270 kg/m^3^, and mineral characterization procedures revealed that BFMW also contains calcite, barite-strontian, fluorite, and quartz [87]. Another type of mine waste is lead mine waste (LMW), which has a high percentage of CaO and other volatiles with 4.65% of Fe_2_O_3_ [88]. LMW has a density of up to 2810 kg/m^3^, with a porosity ranging from 3.06–10.71% [88]. Waste from tin mining, which is known as tin-tailing or amang, has also been used as an aggregate in RSC. Amang has a density of 4000 kg/m^3^, with a high percentage of Fe_2_O_3_ at 46.53% [24]. This waste contains 52.16% of TiO_2_, which is also beneficial for RSC.

#### 3.2.6. Industrial Waste

The utilization of industrial waste could help reduce the volume of waste occupying waste disposal areas. Turkey’s boron industry produced 1.72 million tons of boron minerals and compound in tandem with the amount of waste being produced [89]. Wastes from the iron steel industry, which include steel chips, scale, and slag, have also been used as components in RSC. Steel chips and steel scales contain 60.04% and 67.19% of iron, respectively [90]. These wastes are used as sand replacement of up to 100% in the production of RSC, which showed improvement in shielding against gamma radiation [90,91].

Slags, which are the by-product of iron smelting, are also investigated as a heavyweight aggregate in RSC. Steel slag is one of the common slags used in RSC and is an industrial by-product of various processes in steel production, such as an electric arc furnace, blast furnace, and induction furnace [92]. Steel slag from an electric arc furnace contains 29.01–33.28% of Fe_x_O_y_, which represents the heavy component [73,93]. This type of slag has an apparent density and bulk density of 3854 and 3510 kg/m^3^, respectively, and can be classified as heavyweight aggregate [84,93]. Induction furnace slag contains almost the same amount of Fe_2_O_3_ as an electric arc furnace at 29%, but a higher amount of Al_2_O_3_ [92]. A study using induction furnace slag as the aggregate replacement reported an 18% increment in the density of concrete with 50% replacement, which is 2810 kg/m^3^ [92]. Steel slag from blast furnace slag is commonly used as a cement replacement in RSC and is known as ground granulated blast furnace slag (GGBS) due to its pozzolanic property [17,94,95]. GGBS contains 0.95–3.42% Fe_2_O_3_ and 7.46–15.04% Al_2_O_3_, which are lower compared with other types of steel slag [17,94,95].

Copper slag is another type of slag and is a by-product of copper extraction, with Al_2_O_3_ and Fe_2_O_3_ percentages at 2.87% and 38.37%, respectively [95]. Copper slag has a density of 3800 kg/m^3^. Overall, copper slag possesses a high percentage of heavy elements, such as iron and a high density, which is beneficial to RSC production. Slag from the smelting of lead and zinc ore, known as lead–zinc slag, has also been used in RSC [96,97]. Lead–zinc has a high percentage of nickel at 59.09%, while other elements such as Fe, Al, and Mg are at 11.22%, 6.19%, and 3.48%, respectively [97]. The total replacement of gravel and sand with lead–zinc slag as the aggregate produced concrete with a 30% higher density at 2810 kg/m^3^ [97].

Red mud is another industrial waste that is being studied as an additive in RSC. Red mud is a by-product of aluminum production from bauxite. It contains a high percentage of TiO_2_, Fe_2_O_3_, and Al_2_O_3_ at 20.32–21.2%, 31.88–32.33%, and 7.3–8.5%, respectively [98]. The ceramic form of red mud is produced as a radiation shielding material through sintering and combination with BaCO_3_ or BaSO_4_. However, a high concentration of 232Th and 226Ra in red mud limits its usage in the production of RSC. The presence of naturally occurring radionuclide materials exceeds the world average for the radionuclide concentration in building materials [99].

Boron ores such as colemanite, ulexite, and tincal are the source for boric acid and borate production. The by-product of this industry is borogypsum and colemanite waste that contains boron, which has high a absorption of neutron and is, therefore, beneficial in producing neutron shielding RSC [56,100]. Colemanite waste is a by-product of colemanite concentrating plants, while borogypsum is waste from boric acid plants, which are all part of the boron industry. Colemanite waste and borogypsum contain 6.3–33.99% and 1.05–4.2% of boron, respectively [56,100]. Colemanite and borogypsum wastes have been used for up to 15% of clinker’s weight in the RSC composition [100]. These wastes have also been utilized as fine and coarse aggregates for concrete shielding against electromagnetic waves [56]. Colemanite waste is also used as metakaolin replacement in the production of geopolymers [89], and it improves the microstructure and mechanical properties of geopolymers.

### 3.3. Beneficial Additive

**Figure 4:**
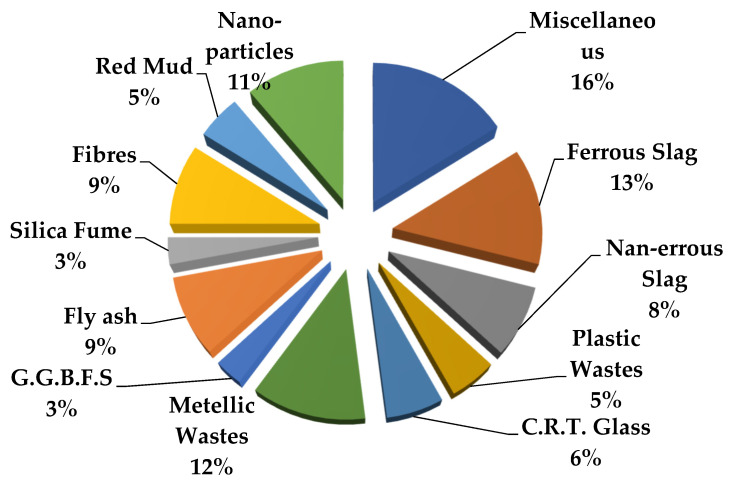
Percentage of the distribution of alternative materials’ usage in RSC.

Various additives are used in concrete for different purposes, such as water-reducing and foaming agents. In RSC, additives increase the shielding performance against gamma ray and neutron radiation. Heavy additives such as nano-TiO_2_ have been used in magnetite RSC by replacing cement content of up to 8% of the weight percentage [101]. Another study used TiO_2_ in comparison to nanosized silica and hematite in barite RSC [22]. The study replaced up to 15% of cement weight with the additive in the mixture. Research on additives of nano-CaCO_3_ and nano-silica in steel slag RSC used up to 3% of the weight of the cement. Research on the neutron absorbing additive of WC and B_4_C used 20% of the total mixture weight [102]. WC is effective in absorbing neutrons due to the presence of carbon, which is similar to boron in the ceramic material of B_4_C. An earlier study used carbon powder of up to 15% of the cement weight in hematite RSC [103]. The study concluded that adding up to 15% carbon powder in hematite concrete has no significant effect on the shielding property. Figure 4 depicts the percentages of several types of alternative materials’ usage in RSC in the most recent literature from 2003 to 2021.

## 4. Radiation Shielding

Radiation is often described in four categories: alpha, beta, X-ray, gamma, and neutron radiation [55]. Radiation from alpha particles is due to the fast movement of the nuclei of the helium atom, while beta particles are from the moving electrons. These two types of radiation can be stopped with a thin sheet of metal because their particles lose energy when colliding with the nuclei of the matter they are passing. X-ray and gamma radiation are electromagnetic waves that can penetrate through matter at a higher degree. Gamma radiation is more intrusive as it has a higher energy than X-ray does [55]. Neutron radiation is due to the emission of neutral particles by atoms that undergo the fission process. Neutron is also highly penetrative depending on its energy and intensity. 

Most research evaluates the performance of RSC in terms of gamma ray and neutron shielding as these types of radiation are more intrusive. Neutron radiation is mostly emitted during the fissioning of atoms, which occurs in the reactor core of a nuclear power generation plant [8]. Gamma radiation is mostly found in spent nuclear fuel [60]. These two types of radiation shielding parameters represent critical parameters in the radiation shielding of RSC and are discussed in the following subsections.

### 4.1. Gamma Ray Shielding

Gamma rays are radiated through radioactive decay and consist of high energy beams that are penetrative. This type of radiation is an electromagnetic radiation that is similar to X-rays but at shorter wavelengths [55]. The high intrusion of gamma rays is further demonstrated through the solidification and removal of micropores in the concrete’s microstructure after 7 days of exposure to a gamma source [104]. The efficiency of RSC in attenuating gamma rays is measured based on the linear attenuation coefficient. It is defined as the probability that a particle in a given material will interact with a photon per unit path length [105]. A linear attenuation coefficient is measured by exposing the RSC sample to gamma rays, which are mostly sourced from Cobalt-60 or Ceasium-137 [12,14,21,63,65,106]. A Cobalt-60-emitted photon has an energy of 1.333 and 1.173 MeV, while Ceasium-137 produces 0.662 MeV [12,14]. 

#### 4.1.1. Test Setup for Gamma Ray Shielding

**Figure 5:**
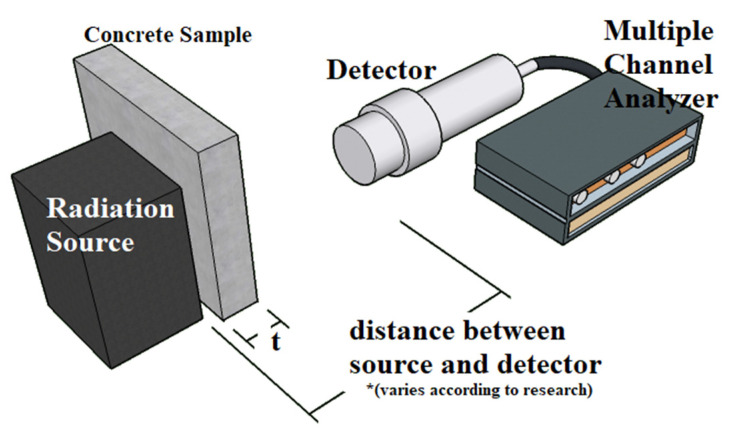
Common linear attenuation coefficient test setup.

The radiation attenuation test setup is shown in Figure 5, where the radiation source is placed tangentially at a certain distance from the sodium iodide (NaI)(Tl) detector, which is connected to multiple channel analyzers (Table 2). Other sources of gamma radiation are used in this study, such as Technetium-99m and Barium-133, which emit 140.511 and 80.99 keV, respectively [107,108]. The distance between the sample to the source and the sample to the detector also varies according to respective research. The shortest distance of a sample to a source of radiation is 20 mm, while the farthest is 790 mm. Therefore, the distance of the source to the detector counter varied from 70–850 mm. However, the distance between the source and detector is insignificant for gamma rays because no absorption of radiation by air occurs due to its high penetration [5]. The selection of the sample’s thickness is either fixed or incremental up to 150 mm. To eliminate the effect of the radiation coefficient damping by specimen thickness, 80–100 mm is recommended by the literature [53,81]. This step is also shown in a study that indicated a test sample with incremental thicknesses, thereby showing that the average coefficient is attained at a 100 mm thickness [41]. Most detector types use NaI(Tl); few studies used a stilbene scintillator to obtain readings for neutron and gamma absorption. In using a stilbene scintillator, the gamma ray counts are measured indirectly by transferring energy to the electrons through Compton’s scattering [109]. The use of this detector allows the measurement of counts for photon and neutron exposure in a single setup of the same source. The duration of the gamma ray counting also varies from as short as 90 s to as long as 120 min. At 120 min and 90 s of measurement, the statistical uncertainty of the data is ±2% and ±0.3%, respectively [62,110]. 

The actual number of counts is obtained by subtracting the background counts in the detector. The background subtracted number of counts with and without a sample are further derived to determine the linear attenuation coefficient based on the Beer–Lambert law, as indicated by Equation (1) [12]:

(1) $$\mu =\frac{1}{t}\ln\frac{D_{0}}{D}$$

where *μ* is the linear attenuation coefficient (cm^−1^), *t* is the thickness of the sample (cm), and *D*_0_ and *D* are the background subtracted number of counts recorded by the detector without and with the RSC sample, respectively. The value of *μ* is mostly the average determined from the plotted relationship between the incremental thickness, *t* of the RSC samples, and the respective value of *μ*.

On the basis of the *μ* value, the value of the half value layer (HVL) and tenth value layer (TVL) can be derived. HVL and TVL values are the thickness required for RSC or any absorbing material to attenuate the intensity of gamma radiation by half and by tenth of its intensity, respectively. HVL and TVL can be calculated using Equations (2) and (3) [118]

(2) $$\mathrm{HVL}=\frac{\ln2}{\mu }$$

(3) $$\mathrm{TVL}=\frac{\ln10}{\mu }$$

where HVL and TVL are the half value layer and tenth value layer, respectively, which are expressed in the unit cm. The mean free path, *mfp*, which is defined as the average distance between two successive photon interactions, can be calculated using Equation (4) [14,17]

(4) $$mfp=\frac{1}{\mu }$$

The linear attenuation coefficient is also expressed in the mass attenuation coefficient as an indicator for shielding the effectiveness by obviating the effect of the varied density in RSC on the *μ* value. The mass attenuation coefficient *μ_ρ_* is calculated using Equation (5) [108,119]

(5) $$\mu_{\rho }=\frac{\mu }{\rho }$$

where *ρ* is the density of the RSC.

Parameters such as HVL, TVL, and *mfp* are derivatives from *μ*, which serves as a gauge for the actual production of material as a radiation shield. Therefore, the linear attenuation coefficient *μ* is the fundamental parameter that determines the efficiency of a material in reducing the energy of nuclear radiation. Further analysis of *μ* into *μ_ρ_* is also useful in gauging the shielding efficiency regardless of its density. Figure 5 shows the common linear attenuation coefficient test setup.

#### 4.1.2. Performance of RSC on Gamma Ray Shielding

The absorption of gamma rays, which is presented by a linear attenuation coefficient, is influenced by the density and atomic number of the shielding material [5]. In RSC, the combination of the binder and aggregates gives RSC a variety of components made of different elements of varied densities. Increasing the amount of high-density elements in RSC increases the absorption rate of the gamma rays. This increase is achieved by increasing the amount of aggregate and by using heavyweight minerals. Increasing the amount of heavyweight aggregate leads to a rise in the density of RSC; RSC with a density more than 2600 kg/m^3^ is categorized as heavyweight concrete [120]. The use of natural aggregates such as limestone and granite, with a density less than 3000 kg/m^3^, indicates the viability of these aggregates to form RSC.

**Figure 6:**
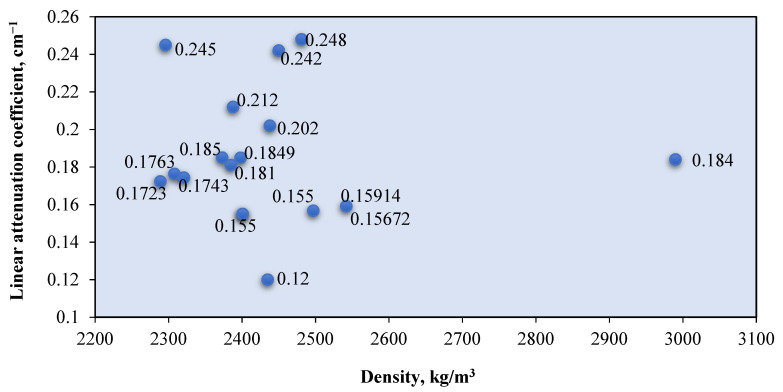
Relationship between linear attenuation coefficient and density of the sample containing natural aggregate (kg/m^3^) (data adapted from [18,24,29,45,53,66,88,95,120,122,123]).

Natural aggregates such as silica sand, granite, and limestone have a density of less than 2800 kg/m^3^ [78,121]. Silica sand and granite contain a large percentage of SiO_2_, while limestone contains mostly CaCO_3_ [72,75,90]. These elements, which are composed of elements with low atomic numbers, resulted in a low gamma attenuation of natural aggregate concrete. The linear attenuation coefficient for various granitic rock is between 0.4–0.455 cm^−1^ [72]. Figure 6 shows the relationship between the *μ* value of the RSC samples that contain natural aggregates from previous research and their respective densities. The *μ* value is taken at an exposure of 0.662 MeV gamma rays on mostly the control sample from each study. The maximum RSC density produced using a natural aggregate is 2990 kg/m^3^, which had a *μ* value of 0.184 cm^−1^ [88]. The maximum *μ* value is achieved by RSC with a density of 2296 kg/m^3^ [95]. A comparison of the mix composition indicates that a higher proportion of natural aggregate in the composition resulted in a higher *μ* value, despite the lower density of concrete. A high amount of aggregate provides a larger percentage of SiO_2_ and CaO in RSC, thereby leading to a higher *μ* value. On the basis of the data presented in Figure 3, the average density of natural aggregate RSC is 2434.87 kg/m^3^ with a *μ* value of 0.1855 cm^−1^, which equals to the mass attenuation coefficient *μ_ρ_* of 0.0762 cm^−2^/g.

**Figure 7:**
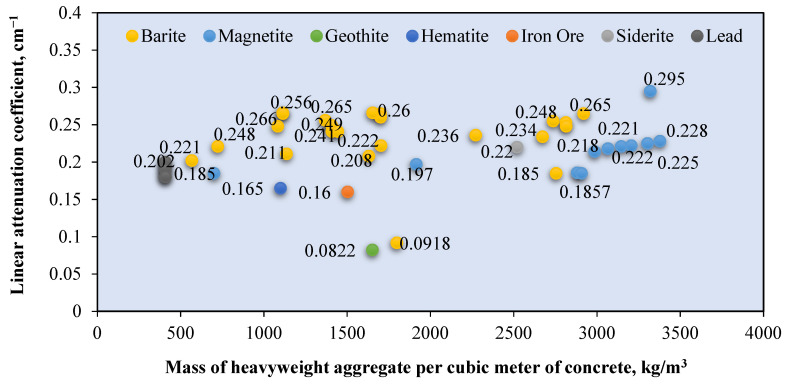
Relationship between linear attenuation coefficient (cm^−1^) and the amount of heavyweight minerals in concrete (kg/m^3^) (data adapted from [12,14,18,29,44,45,53,63,67,88,106,120,123]).

A naturally occurring heavyweight aggregate has a bulk density of more 3000 kg/m^3^ and includes barite, magnetite, hematite, limonite goethite, and ilmenite [76]. Figure 7 shows the relationship between the *μ* value and mass of the heavyweight aggregate per cubic meter of RSC. The value of *μ* is recorded based on exposure to gamma rays at 0.662 MeV. Recent research mostly used barite and magnetite in investigating RSC, as shown in Figure 4. Few studies used hematite, iron ore, siderite, and lead in the composition of RSC and recorded a maximum *μ* value of 0.22 cm^−1^ [18,63,67,120,123]. 

Barite has a density of 4000 to 4200 kg/m^3^ and contains a large percentage of barium sulfate (BaSO_4_); this percentage varies according to its purity [17,76,77]. A combination of coarse and fine barite aggregate with a high water-to-cement ratio resulted in 3950 kg/m^3^ for RSC’s density [88]. The linear attenuation coefficient of barite ore with 90% BaSO_4_ is around 0.75 cm^−1^ [35]. From Figure 4, RSC containing barite has an average *μ* value of 0.244 cm^−1^, which is 31.3% higher than the *μ* value reported for natural aggregate RSC. The heavy element of barium in barite aggregate resulted in superior shielding to that provided by silicon or calcium in natural aggregate. General observation also indicates that the increase in the amount of barite aggregate in a sample caused an increment in the shielding value. On the basis of the available RSC density data, the average density of barite RSC is 3366.121 kg/m^3^ and, hence, a *μ_ρ_* value of 0.066 cm^2^/g. The presence of barium in BaSO_4_ compounds influences the high absorption value of barite RSC. Hence, a high purity of barite aggregate would result in a high *μ* value.

For magnetite RSC, the highest recorded *μ* value is 0.295 cm^−1^ for a sample that contained only coarse and fine-sized magnetite at 3320 kg/m^3^ [53]. However, a lower *μ* value of 0.228 cm^−1^ was recorded for a sample that contained a slightly higher magnetite mass of 3378.3 kg per cubic meter of concrete [12]. This result may be due to the lower purity of magnetite used in the study, which reported a percentage of Fe_3_O_4_ at 72.1–78.2% compared with 90% in the study of Horszczaruk and Brzozowski (2019) [12,53], who examined the effect of the water-to-cement ratio on the shielding properties of magnetite concrete and indicated that the *μ* value is reduced due to an increase in the water-to-cement ratio of the concrete [12]. However, on the basis of the mixed composition of the samples, the amount of magnetite aggregate is also gradually reduced across the samples, apart from the increment in the water-to-cement ratio of the sample. This finding shows that the reduction in the *μ* value may also be due to the reduced magnetite aggregate in the RSC. This result is also shown by samples that contained only 700 kg/m^3^ of magnetite and had the lowest *μ* value of 0.185 cm^−1^ [106]. The data in Figure 4 show that the average density of magnetite concrete is 3618.7 kg/m^3^, which is derived from the *μ_ρ_* value of 0.060 cm^2^/g. The presence of iron as a heavy element in magnetite influences the absorption value of the produced RSC and depends on the purity of the magnetite aggregate used in the concrete.

Hematite ore contains a similar heavy element of iron, which contributes to a higher radiation shielding value. A hematite ore sample containing 27% Fe_2_O_3_ had a *μ_ρ_* value of 0.263 cm^2^/g at 0.081 MeV of radiation [28]. This result is 687% and 97% lower than that of barite and magnetite ore, respectively. This finding indicates that gamma attenuation is largely influenced by the percentage of the heavy element in aggregate as barite and magnetite contained 93.1% of BaSO_4_ and 85.74% of FeO, respectively. Hematite RSC had a *μ* value of 0.165 cm^−1^ at 0.662 MeV of radiation [18]. This is produced by hematite with 71.71% of Fe_2_O_3_. Another study on hematite RSC reported a 0.189 and 0.160 cm^−1^ *μ* value at 0.662 and 1.25 MeV, respectively [124]. A slightly higher *μ* was reported at 0.212 cm^−1^ for 0.662 MeV exposure to Cs-137 [103].

Ilmenite RSC contains titanium and iron as the heavy element, with 35.55–65.74% Fe_2_O_3_ and 21–23.08% TiO_2_ with a specific gravity of 4.2 [79,80]. This resulted in an attenuation coefficient percentage of 9.81%, which is higher than that of barite RSC at 9.73% [80]. This result is based on exposure to 660 keV of photon energy, and the resulting attenuation coefficient percentage is largely influenced by the higher density of ilmenite used in the study compared with barite and gravel.

**Figure 8:**
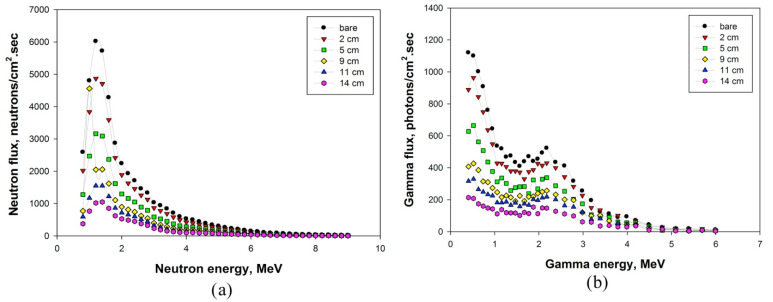
(**a**) Quick neutron spectra in dolomite concrete at different thicknesses. (**b**) Total ray spectra in dolomite concrete at different thicknesses [67].

For limonite RSC, a study that used limonite ores with 70.94% of Fe_2_O_3_ had a *μ_ρ_* value of 0.368 cm^2^/g, which is 3.4% higher than that of hematite, but 29.2% lower than that of magnetite [28]. For goethite RSC, a mix of goethite as the coarse aggregate with limonite and natural sand as the fine aggregates resulted in a *μ* value of 0.0822 cm^−1^ (Figure 8) [67]. The study shows that the goethite aggregate used in the sample contained 67% Fe_2_O_3_, which is high, yet the *μ* value is 12% lower than that of barite RSC with limonite and sand as the fine aggregates. The barite in the RSC sample had a slightly lower percentage of barium oxide at 66.77%, possibly because of the larger atomic number of barium compared with iron, which resulted in a larger percentage of shielding. The slope of the attenuation curves (Figure 8) was used to calculate the integrated fast neutrons’ discharge macroscopic cross section (Σ) and the average values of the linear attenuation coefficient (*µ*) for the total gamma rays (primary and secondary). The HVL for each concrete mix for both fast neutrons and total gamma rays was calculated using the mathematical equations developed by Baratta [125].

Another type of aggregate called zeolite, which contains alumina silicate, has also been studied as an aggregate for RSC. Zeolite contains 72.09% SiO_2_ and 13.612% Al_2_O_3_ [126]. Increasing the percentage of zeolite, replacing the natural aggregate up to 50%, resulted in the reduction of the *μ* value by about 40% [38]. The lowest *μ* value at 0.156 cm^−1^ was recorded by the 50% concentration of the zeolite aggregate and was due to the high porosity of zeolite, hence endowing it with a lower density and shielding property. Therefore, the study concluded that zeolite is not a viable aggregate replacement in RSC. Table 3 summarizes the comparison between various studies on different aggregates used in RSC. A comparison between a sample with high purity and one with a lower purity of barite indicates a reduction in the *μ* value; this is also shown in the varied purity of magnetite and other types of ore such as hematite and goethite. 

Overall, the element that constitutes the aggregate heavily influences the outcome of the shielding efficiency. The purity of the ores used as the aggregate determined the percentage of heavy elements possessed by the mineral, hence affecting the *μ* value of the concrete.

Various sizes of heavyweight or natural aggregates were used, and this influences the *μ* value of RS. Research on magnetite RSC shows a 12% increase in the *μ* value with an increase in the magnetite maximum aggregate size from 12.5 to 25 mm [127]. This result of *μ* is based on gamma ray radiation at 0.662 MeV. This is also indicated by barite concrete, with the highest value of *μ* at 0.266 cm^−1^ and a larger proportion of a coarse-size aggregate compared with a fine aggregate [29]. One study that used a fine-sized barite aggregate reported a lower *μ* value of 0.241 cm^−1^ at 0.662 MeV of gamma radiation [45].

Another study that used fine-sized barite as the aggregate reported an even lower *μ* value of 0.208 cm^−1^ at a similar energy level [18]. A comparison among samples with a natural aggregate also indicates the influence of aggregate sizes on the radiation shielding efficiency. A sample with a coarse-to-fine aggregate ratio of 1.6 had a higher *μ* compared with samples with a coarse-to-fine aggregate ratio of 0.8–1.18 [29,66,123]. This result may be due to the higher surface area of a fine-sized aggregate compared with that of a coarse aggregate, which created a larger interfacial transition zone. The increase in the interfacial transition zone leads to the reduction in the *μ* value. In terms of the variation in the water-to-cement ratio of RSC, no significant impact on the *μ* value is observed. This finding is shown by the natural aggregate concrete sample with a water-to-cement ratio of 0.16 having an almost similar *μ* value as a sample with a 0.4 ratio. This result is further proved by samples with similar constituents but a varied water-to-cement ratio of 0.43–0.63. The samples showed no significant changes in the *μ* value, which was around 0.19 cm^−1^ [113]. 

**Figure 9:**
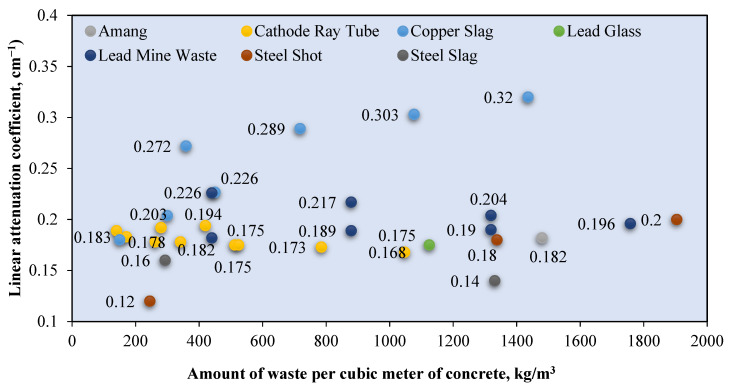
Relationship between linear attenuation coefficient (cm^−1^) and amount of waste per cubic meter of concrete (kg/m^3^) (data adapted from [14,24,65,88,95,106,120,122]).

Industrial waste has also been utilized as RSC. This approach produces more sustainable concrete by reducing waste and reduces dependency on naturally occurring minerals such as barite and magnetite. Figure 9 shows the reported *μ* value for different types and masses of waste incorporated per cubic meter of concrete. The reported *μ* value is based on exposure to gamma radiation at 0.662 MeV. Most data indicate that waste is utilized at about 100–1500 kg/m^3^ of the RSC mixture (Figure 5). A sample that consists of only a steel shot as the aggregate and dune sand as the fine aggregate had a linear attenuation coefficient of 0.2 cm^−1^ [120]. This value is higher than that of a sample with steel slag or a combination of coarse limestone and a steel shot. As a result, it is found that the highest amount of steel shots in mix constituent produced concrete with the highest unit weight, and hence the highest *μ* value. The *μ* value is 3% higher than the maximum value exhibited by the magnetite concrete with 420 kg/m^3^ of a cathode-ray tube [106]. This finding implies that the improvement in the *μ* value due to steel shots as the aggregate is comparable to that of a heavyweight aggregate such as magnetite. The replacement of heavy minerals with a cathode-ray tube in RSC reduces the weight and hence achieves a *μ* value of as low as 0.168 cm^−1^ [106]. The utilization of waste containing lead glass had a *μ* value of 0.175 cm^−1^, which is about the value of the cathode-ray tube RSC as this material contained mostly SiO_2_. The by-products of the mining process, such as amang and lead mine waste, had higher *μ* values of 0.182 and 0.175 cm^−1^, respectively [24,88]. 

A higher *μ* value in RSC with amang compared with that of lead mine waste RSC is due to the presence of TiO_2_ and Fe_2_O_3_, which are heavier elements than CaO and make up most of the lead mine waste’s composition. Copper slag RSC has the highest *μ* value of 0.32 cm^−1^ [95]. The study indicates that copper slag as a fine aggregate combined with the coarse natural aggregate increased the *μ* value by 30.6%. The utilization of lead slag recorded a lower coefficient of 0.204 cm^−1^ at a 60% aggregate replacement [128]. The study indicates that the replacement of the aggregate with lead slag from 40% to 60% resulted in a 5% increase in *μ*. This result is in line with a study on the effect of lead–zinc slag as a fine aggregate in RSC, which shows a 17% improvement in the *μ* value with the incorporation of slag [97]. However, this condition is opposite to the steel slag performance in RSC because the increment in the steel slag proportion resulted in a reduced *μ*, which may be due to the lack of heavy elements in this type of slag [120]. 

Other research on the replacement of steel shots with electric arc furnace slag also indicated a reduction in *μ* with an increasing replacement amount [84]. At a maximum replacement of 65% of steel shot with slag, the density is 14.6% lower than that of concrete with 100% steel shot as the aggregate. This result shows that the density of slag is lower than that of steel shots due to the lack of heavy elements in its composition, hence resulting in a lower attenuation coefficient. Mostly, copper and steel slag consist of Fe_2_O_3_ at different percentages and some steel slag is reported to contain Al_2_O_3_ [93,95,129]. Lead–zinc slag contains mostly nickel, which is heavier than iron, thus explaining the high recorded *μ* value [97]. The type and amount of element influence the efficiency of the overall gamma radiation attenuation, which could enable slag to perform similarly to heavyweight minerals. This finding is shown by a study on a sample with electric arc furnace slag as the aggregate in the RSC that had a similar *μ* value as barite RSC of 0.182 cm^−1^, based on exposure of 1.25 MeV [129]. The slag aggregate is denser than the barite aggregate because of the high amount of magnetite and hematite in the EAF, which contributes Fe_x_O_y_ elements and thus results in high gamma ray attenuation. The variations in the composition of industrial waste affect the shielding coefficients, which could outperform heavyweight aggregates, hence producing more sustainable RSC.

**Figure 10:**
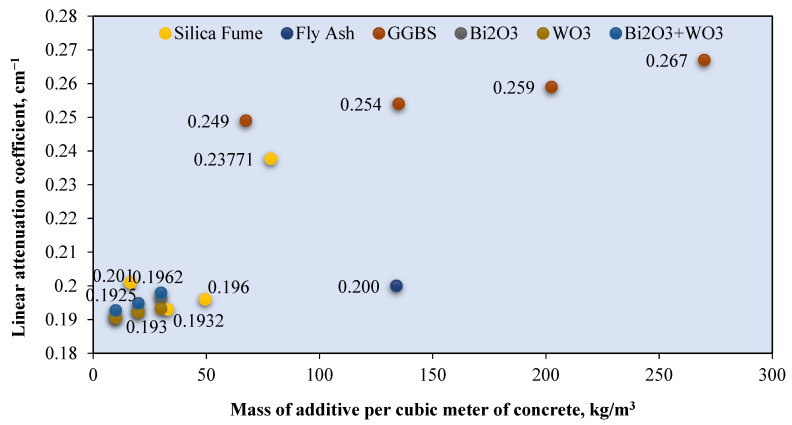
Relationship between linear attenuation coefficient (cm^−1^) and amount of additives in concrete (kg/m^3^) (data adapted from [14,44,63,95,122,128]).

Few gamma ray attenuation studies investigated the effect of a pozzolanic additive, such as silica fume and GGBS, and also other heavyweight additives such as Bi_2_O_3_ on the linear attenuation coefficient. This effect is shown by Figure 10, which exhibits the relationship between the *μ* value (cm^−1^) and the weight of the additive added per cubic meter of RSC (kg/m^3^). The *μ* value shown in the figure is based on exposure to photons at a 0.662 MeV energy level. Additives such as silica fume [130], GGBS [131,132,133,134], rice husk ash [135,136], palm oil fuel ash [137,138,139,140,141], and fly ash [142,143,144,145,146,147,148,149,150,151] serve as a partial cement replacement, which aims to reduce the carbon footprint of concrete [152,153,154,155,156,157]. The 60% increase in the fly ash replacement for cement in concrete reduced the *μ* value by 10% [63]. The study also reported no reduction in *μ* for 20% fly ash replacement for cement, which indicates the optimum percentage. This result is in line with other research that limited the percentage of fly ash in the mix design by 20% [65,106,158]. Silica fume is added to RSC at 19.5–23.1% of the total binder weight [18,159]. A 13% increment in the percentage of silica fume-to-total binders resulted in the reduction of the *μ* value between 2.5–4.3% [128]. 

Another study that used 2.8% silica fume in RSC also reported a reduction in the gamma ray attenuation coefficient [41]. This result is due to the lower density of silica fume compared with that of cement, which resulted in a lower RSC density and *μ* value. For GGBS, the replacement of cement with GGBS by 15–60% resulted in a 1.6–9.0% increment in the *μ* value [95]. This result is due to the micro-filling ability and pozzolanic reactivity of GGBS, which leads to densification of the pore structure and, hence, better attenuation [95]. Additives with a high atomic weight such as Bi_2_O_3_ have also been used in improving RSC. The use of Bi_2_O_3_ in RSC showed a 2.6–5.7% increment in the *μ* value, with a 2–6% replacement for cement [14]. The 2–4.0% WiO_3_ replacement for cement increased the *µ* value [14]. The gain recorded by Bi_2_O_3_ is higher than that of WiO_3_ because the latter has a lower density due to the lower atomic number of the elements in the component [14]. This condition resulted in a higher density of concrete due to the presence of Bi_2_O_3_ compared with WiO_3_. The combination of the two additives at a 2–6% replacement resulted in a slightly higher gain of 3.7–6.6% in the *μ* value. This result is due to the compound effect of both additives on neutron and photon absorption [14]. In the overall observation, the *μ* value recorded by these additives is below 0.2 cm^−1^, which is lower than that of concrete with the GGBS additive, as shown in Figure 5. However, the percentage of the *μ* increment for each percentage of additives is larger for the Bi_2_O_3_ and WiO_3_ combination compared with GGBS. This finding further shows the influence of an element with a high atomic number on the attenuation property. 

By linking the findings of each research, it is notable that the presence of elements with a high atomic number and the improving microstructure of concrete resulted in a higher shielding performance. Incorporating additives into the RSC composition affects its microstructure. Pozzolanic additives provide additional calcium silicate hydrate in concrete’s matrix through a secondary pozzolanic reaction, while heavyweight fillers increase the radiation attenuation property. These changes in concrete’s constituent and microstructure, which aimed to improve the shielding properties of concrete, also affect its mechanical strength, which is the governing parameter in defining its practicality as a structural member.

### 4.2. Neutron Shielding

Neutrons are uncharged particles released by fissioning atoms, and shielding against neutrons involves capturing these particles [3]. Fast-moving neutrons need to be slowed down through inelastic collision with heavy elements such as lead, and their velocity can be reduced further by collision with a light element such as hydrogen [5]. The principle in measuring the shielding against gamma rays is also used in neutron shielding.

#### 4.2.1. Test Setup for Neutron Shielding 

The test setup for irradiating RSC by using a neutron source is similar to that shown in Figure 1; the only difference is the type of scintillating detector to detect neutron flux. For neutron radiation, radiation sources can either be a plutonium–beryllium source (Pu–Be) or a americium–beryllium source (Am–Be). Pu–Be emits energy of 3.92 and 4.43 MeV, while Am–Be emits about 4.5 MeV [110,160]. As neutrons have no charge, their detection is performed indirectly through nuclear reactions that produce charged particles [161]. The detector produces pulses based on the neutron reactions within it. 

The total intensity of neutrons with and without RSC is used to determine the total neutron cross section or macroscopic removal cross section ∑_R_ (E_n_) in cm^−1^, which can be calculated using Equation (6) [110,160]

(6) $$\sum_{R}\left(E_{n}\right)=\frac{1}{t}\ln\frac{I_{0}}{I}$$

where *t* is the thickness of sample (cm), and *I_0_* and *I* are the total intensity of the fast neutron flux emitted without and with the sample, respectively. The total macroscopic removal cross section, ∑_R_ (E_n_), is the probability of a fission or fast neutron having a first collision that would displace it from the group of penetrating neutrons [162]. A high value of ∑_R_ (E_n_) corresponds to a large number of neutron collisions, which is equivalent to the absorption of neutrons. This value depends on the target nucleus of the shielding material and also the energy possessed by the neutrons. High-energy neutrons called fast neutrons are attenuated by elastic collisions with nuclei of light elements such as hydrogen. The attenuation is also caused by inelastic collisions with nuclei of heavy elements [163]. These slowed-down neutrons, called thermal neutrons, are captured by the absorber atom in compounds such as boron or gadolinium [3,163]. 

Similar to gamma ray attenuation, ∑_R_ (E_n_) is used to determine HVL and relaxation length *λ* using Equations (7) and (8) [110]

(7) $$\mathrm{HVL}=\frac{\ln2}{\;\sum_{R}\left(E_{n}\right)}$$

(8) $$\lambda =\frac{1}{\sum_{R}\left(E_{n}\right)}$$

#### 4.2.2. Performance of RSC on Neutron Shielding

Table 4 summarizes the value of the macroscopic removal cross section for the RSC sample from previous studies. The highest ∑_R_ (E_n_) value recorded by RSC with a light natural aggregate is 0.133 cm^−1^, based on exposure to 0.025 MeV of energy [123]. Concrete with granite aggregate recorded a lower value of ∑_R_ (E_n_) at 0.104 when exposed to a higher energy level of 4 MeV [160]. Another study that used a natural aggregate in the RSC recorded a lower ∑_R_ (E_n_) value of 0.075 cm^−1^ at 4 MeV [13]. On the basis of the mix composition of RSC used in the studies, the increase in the amount of cement used in the sample resulted in a gain in neutron shielding. This gain was achieved because chemically bound water in cement hydrates that contain hydrogen has a vital role in slowing down fast neutrons [109]. Another study agrees with this finding, indicating that a 1% increment in the cement’s moisture resulted in a 15% increment in the ∑_R_ (E_n_) value [164]. 

The heavyweight aggregate provides heavy elements, which cause inelastic collision with neutrons. This collision precipitates energy and affects the neutron shielding property. A study on magnetite RSC indicates that a heavyweight aggregate is not effective against fast neutrons because of the lower number of thermal neutrons after passing through the magnetite RSC sample compared with the granite RSC sample [164]. 

However, the heavyweight aggregate absorbed a higher percentage of thermal neutrons at 90% compared with the granite sample at 70% absorption. This finding is also shown by another study that reported a 16–20% lower HVL of magnetite RSC compared with granite concrete [163]. However, the study attributed the superior performance of magnetite compared with granite to the better attenuation of fast neutrons by iron in magnetite compared with silica in granite. The positive impact of the heavyweight aggregate on neutron shielding is also shown by barite. The barite RSC recorded a lower dose transmission of 0.862 compared with 0.864 recorded by the natural aggregate concrete [161]. This reduction in the neutron dose is also translated in terms of ∑_R_ (E_n_), where the barite concrete had a higher value of 0.15 cm^−1^ compared with the natural aggregate concrete at 0.13 cm^−1^ [123]. 

However, a study on the replacement of limestone with hematite revealed no significant impact of the hematite presence on the neutron shielding properties of the concrete sample [81]. A concrete sample with a 50% hematite replacement for the limestone aggregate recorded a ∑_R_ (E_n_) value of 0.14112 cm^−1^, which is a marginal increase compared with the 100% limestone concrete at 0.13954 cm^−1^.

**Figure 11:**
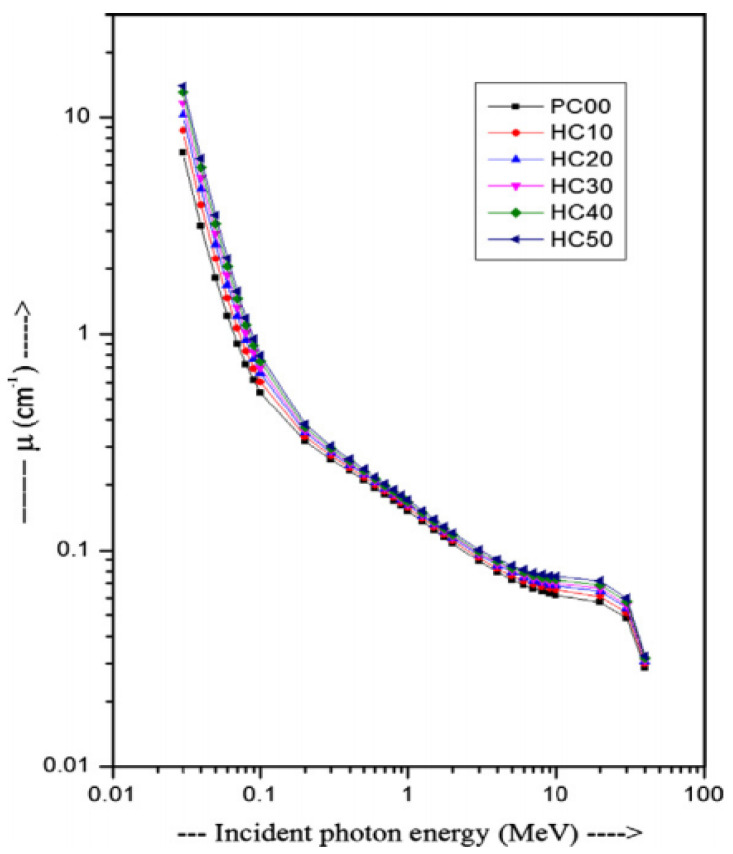
Variation of attenuation coefficient with photon energy for various samples [81]. Annotations: Hematite proportion: 50% (HC50), 40% (HC40%), 30% (HC30), 20% (HC20), and 10% (HC10).

This result may be due to the low amount of heavy elements in hematite and the low percentage of replacement. Overall, the presence of heavy elements in the aggregate caused energy precipitation due to inelastic collision. However, the moderation of fast neutrons due to inelastic collision is not very effective, which results in a slight gain in ∑_R_ (E_n_). Figure 11 shows the gamma linear attenuation coefficient values for six separate samples [81]. The linear attenuation, which is clearly relevant for radiation shielding, generally explains the liability of an incoming photon mixing with a certain material per unit of travel length. The attenuation is proportional to the density of the material.

The presence of light elements such as hydrogen provides a cross section for the elastic collision with neutrons, which contributes to the overall increment in ∑_R_ (E_n_). Colemanite is a type of mineral that contains hydrogen, whose monoclinic crystal structure has a chemical formula of 2CaO.3B_2_O_3_.5H_2_O, and boron, which makes colemanite the prime source for borax and boric acid production [82]. Boron atoms have a large cross section, which is beneficial for neutron absorption and interaction [167]. Hence, 0.25–15% of colemanite was included in concrete’s mix constituent in previous studies [77,116]. A study revealed that a 15% colemanite replacement for the natural aggregate and barite aggregate in concrete resulted in 1% and 1.7% reductions in neutron dose transmission, respectively. Concrete with the natural aggregate replaced with 15% colemanite recorded a neutron dose transmission of 0.855; while concrete with the barite aggregate replaced with 15% colemanite recorded a lower neutron dose transmission of 0.846. This finding indicates the compounded effect of the heavyweight aggregate and colemanite in absorbing neutrons.

Serpentine also contains hydrogen as it is a hydrate aggregate that retains water crystallization at temperatures up to 500 °C [119]. Serpentine is a metamorphic mineral with the chemical formula of Mg_3_Si_2_O_5_(OH)_4_. Serpentine concrete recorded ∑_R_ (E_n_) of 0.0922 cm^−1^ at 4 MeV exposure, which was contributed largely by chemically bound water in serpentine [109]. The combination of serpentine and heavyweight minerals as the aggregate in concrete has also been studied. A study on serpentine replacement with barite or hematite as the aggregate was conducted with exposure to Pu–Be at an energy level of 0.8–11 Mev [110]. This finding indicates that replacing serpentine with 50% barite achieved the highest ∑_R_ (E_n_) of 0.1484 cm^−1^. This value is higher than the ∑_R_ (E_n_) value recorded for 50% of hematite replacing serpentine at 0.1398 cm^−1^. Both types of aggregate replacement showed a 51–61% gain in the ∑_R_ (E_n_) value compared to the control sample of serpentine concrete because of the inelastic collision of neutrons and heavy elements, contributing to the numbers of moderately fast neutrons, which were also caused by the elastic collision of neutrons with hydrogen.

Limonite is another type of hydrated aggregate with the chemical formula 2Fe_2_O_3_.3H_2_O [76]. The replacement of the normal aggregate with limonite in concrete increased the effective macroscopic removal cross section ∑_R_ [165]. The highest ∑_R_ value of 0.148 cm^−1^ was recorded for 100% replacement of natural aggregate with limonite. This result occurred because of the highest partial density value of hydrogen in the limonite concrete sample, which was contributed by crystallized water in the limonite aggregate. Overall, the presence of the neutron moderator, such as the hydrogen, and neutron absorber, such as boron, improves the shielding of RSC against neutrons. As shown in previous findings, samples with these elements recorded a higher value of ∑_R_ (E_n_).

The incorporation of the peridotite aggregate in concrete resulted in an almost 50% increment in the neutron attenuation rate compared with ordinary concrete [166]. The peridotite aggregate contained mostly MgO and SiO_2_, but also 12.95% of crystallized water. On the basis of Am–Be exposure, peridotite concrete recorded a ∑_R_ value of 0.1445 cm^−1^, which is almost 50% higher than that of ordinary concrete at 0.0671 cm^−1^. The presence of crystallized water in peridotite concrete provides hydrogen with a high cross section and thus, a higher removal cross section value. 

Neutron-absorbing elements such as hydrogen and boron are integral in producing neutron shielding concrete. Other materials with a hydrogen source such as styrene butadiene (SBR) and high-density polyethylene (HDPE) have been studied as aggregates in concrete. SBR is a latex polymer that can act as a binder and can be molded into various shapes. It has been used to form shielding material by adding lead oxide or lead ore mineral to the elastomer matrix [168,169]. Recent research has used SBR as a 10–20% cement replacement to increase the amount of hydrogen in concrete [13,64]. Concrete with a 15% SBR replacement of cement resulted in a maximum ∑_R_ (E_n_) value of 0.09 cm^−1^ [13]. This result is based on Am–Be exposure at a 4 MeV energy level. In terms of the dose transmission value, the increase in hydrogen loading due to SBR replacing cement reduces the dose transmission value by 5.3% on average. This improvement in neutron shielding due to the increase in the hydrogen presence in concrete is caused by a scattering reaction, which is facilitated by the hydrogen, hence reducing the transmission value [13]. In the utilization of HDPE as a concrete additive, a concrete sample containing HDPE and a natural aggregate had a ∑_R_ (E_n_) of 0.108–0.11 cm^−1^, which is higher than that of SBR [160]. This result is due to the amount of hydrogen loading in the HDPE concrete mix being higher by 217% compared with that of the SBR concrete mix. 

**Figure 12:**
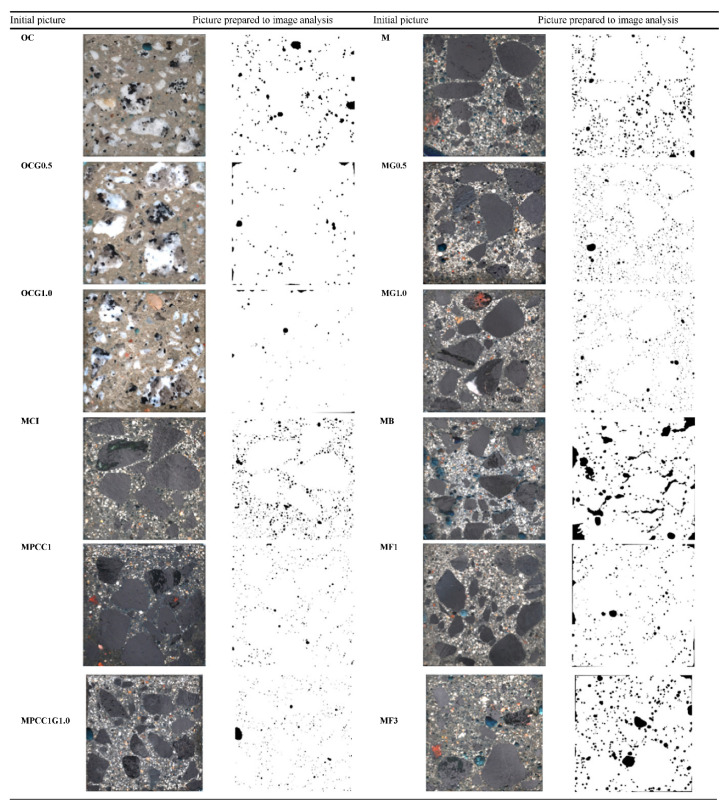
Mean porosity (V_v_) computed by image analysis with variation coefficients (CV, CV_2_), and without/with air void contribution of ≥2 mm (V_v2_) (adapted from [163]).

In comparison to the control sample, a 50.4% replacement of fine aggregates with HDPE resulted in a 5.7% increase in the ∑_R_ (E_n_) value, while a 15% cement replacement with SBR resulted in a 20% increment in the ∑_R_ (E_n_) value (Figure 12). ∑_R_ (E_n_) increased with the addition of acrylic and gadolinium [163]. Acrylic is a polymer dispersion that is rich in hydrogen, while gadolinium has the highest thermal neutron capture cross section. With the 10% addition of acrylic and 1% addition of gadolinium of the cement mass in the concrete mix, 5.99 cm of measured HVL and 0.1034 cm^−1^ of calculated ∑_R_ (E_n_) were obtained [163]. 

Figure 12 shows that the porosity V_v_ computed by image analysis for normal and magnetite concrete reduced as the gadolinium content increased [163]. In general, high-density concrete with acrylic dispersion (MPCC1), normal concrete with 1.0% gadolinium oxide (OCG1.0), and high-density concrete with acrylic dispersion and 1.0% gadolinium have the lowest porosity (between 1.1% and 1.2%). A mix of micro- and macrofibers had the highest V_v_ (MF3, 6.45%) and magnetite concrete with sodium borohydride (MB, 6.81%). This condition is most likely the result of the addition of a high volume of fibers in a mix, resulting in a decreased workability [170].

Aside from being incorporated in concrete via colemanite, boron is also included in concrete through infusion with heavyweight ores or as an additive through a material that contains boron. In a study that used barite as a heavyweight fiber in normal concrete, boron oxide is infused with the barite fiber to improve the neutron shielding property of concrete [158]. An infused barite fiber that contains 12% B_2_O_3_ was added to the concrete composition at 5 kg per cubic meter. Exposure to the Pu–Be neutron source revealed a 25% increase in neutron shielding properties for a sample thickness of 10 cm with a current transmission rate of 0.48. 

This increment in shielding is based on a comparison with normal concrete without a barite-infused fiber, which had a higher current transmission rate of about 0.62. Boron wastes were also studied as an additive in concrete through the usage of borogypsum and colemanite wastes. These boron wastes are by-products of boric acid and borate production [56]. Borogypsum and colemanite wastes contained 1.05–4.2% and 6.3–33.99% B_2_O_3_, respectively. 

A study that used borogypsum and colemanite waste as an additive in concrete indicated an increase in the shielding of the electromagnetic wave and photon ray [56,100]. Another type of boron source, which is boron carbide, also achieved improved neutron shielding with its presence. An 11.43% addition of boron carbide of the overall magnetite concrete weight resulted in a 20% increase of the ∑_R_ of fast neutrons to 0.128 cm^−1^ [64]. In terms of the slow neutron cross section, magnetite concrete with boron carbide recorded a ∑_R_ of 0.196 cm^−1^, which is a 35% increment compared with that of normal concrete. These neutron shielding values were measured in a study that used the research reactor ET-RR-1. Boron carbide is a ceramic material used as an additive in nuclear protective material and is a good slow neutron absorber as it contains a ^10^B atom. ^10^B has a high cross section of 3837 barns to capture thermal neutrons [86,171].

## 5. Mechanical Strength Properties

The practicality of concrete as a structural member is defined by its mechanical strengths, such as compressive, tensile, and flexural strength. Alterations to concrete’s composition to improve its radiation shielding properties affect these strengths. 

### 5.1. Compressive Strength 

**Figure 13:**
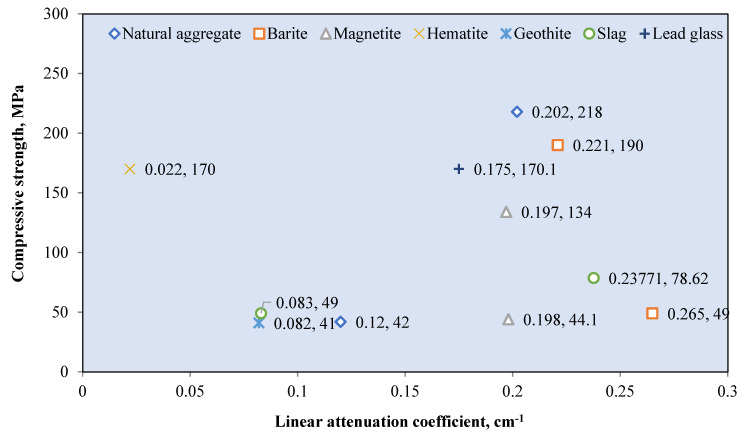
Compressive strength and linear attenuation coefficient of various RSCs (data adapted from [14,24,44,45,46,53,67,120,122]).

Figure 13 shows the compressive strength of RSC from previous research along with the value of the linear attenuation coefficient. The highest compressive strength recorded for RSC is 218 MPa, which consists of natural aggregates, and has a *μ* value of 0.202 cm^−1^ [45]. This type of concrete has a compressive strength above 180 MPa and is considered UHPC [172,173]. A high compressive strength is achieved by using a very low water-to-cement ratio of less than 0.2 to reduce free water in the concrete paste and fewer pores in the hardened concrete [44,45,174,175]. 

The replacement of the natural aggregate with barite in UHPC concrete resulted in a 12.8–21.1% reduction in compressive strength [18,45]. This result is due to the lack of enhancement in the interfacial transition zone between barite and cement paste and the disintegration of barite during the mixing process [45,74]. The use of magnetite in UHPC also achieved a 12.9% reduction in compressive strength compared with the natural aggregate [44]. 

However, at a coarser size of the magnetite aggregate, a 6.7% lower reduction in compressive strength was recorded compared with that of the natural aggregate [53]. Goethite concrete had a higher compressive strength compared with barite concrete, but was lower than that of the natural aggregate concrete.

**Figure 14:**
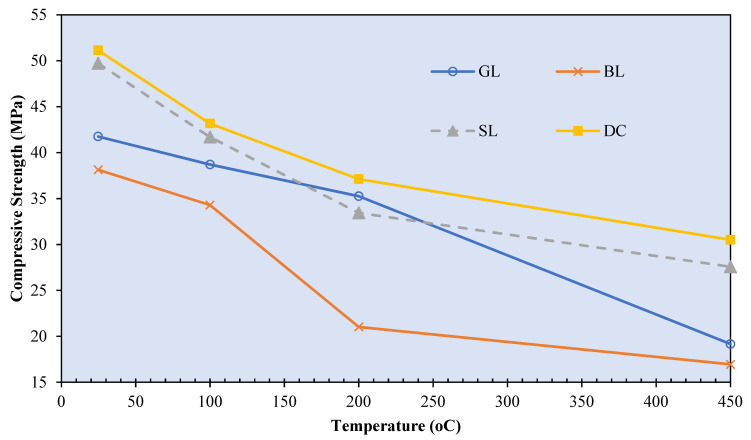
Compressive strength for preheated and unheated concrete at varying temperatures (adapted from [67]).

At a similar concrete density, goethite concrete had 41 MPa of compressive strength, which is 3 MPa higher than that of barite concrete, but 10 MPa lower than that of natural aggregate concrete (Figure 14) [67]. However, a study shows that the replacement of the natural aggregate with hematite powder resulted in a 6.3% increase in compressive strength [46], which is achieved at a 50% replacement of dune sand with hematite powder, and the sample reported 170 MPa of compressive strength. This increase is due to the very fine size of hematite powder, which resulted in a higher packing density and its angular morphology, thereby increasing the interlocking strength between components of the concrete [46].

Colemanite replacement in ordinary concrete achieved a 14.51–51.61% reduction in compressive strength. The reduction is due to the replacement of the natural aggregate by 10–50% of the volume with colemanite [176]. The highest compressive strength of concrete with colemanite is 50 MPa at 10% replacement of the aggregate. This condition is also exhibited by barite concrete with colemanite replacement, which showed 33.33% and 46.67% reductions in compressive strength due to a 5% and 10% replacement, respectively [167]. The adverse effect of the presence of colemanite on the compressive strength of concrete is due to the reaction between cement paste and colemanite, which resulted in melting and flocculation. This flocculation led to the formation of deep and wide cracks at the interfacial zone between the colemanite and cement paste, hence weakening the adhesion and lowering the overall compressive strength [176]. 

This weakness at the interfacial transition zone is also reported for goethite and serpentine concrete, which leads to the reduced compressive strength of the sample. The use of goethite as a coarse aggregate resulted in a compressive strength of 39–42 MPa, which is about 38% lower than that of magnetite concrete [17]. For concrete with serpentine aggregate, the compressive strength is even lower, at around 32 MPa. Researchers noted that the serpentine and goethite coarse aggregates have a high water absorption rate, which causes internal bleeding at the aggregate surface. This condition leads to the formation of a porous interfacial transition zone, hence weakening the bond between the aggregates and concrete matrix [17].

Waste as the aggregate in concrete has a varied impact on the compressive strength. This condition depends on the physical and mechanical properties of waste. The total replacement of the fine barite aggregate with a cathode-ray tube resulted in a 29% reduction in compressive strength [65]. This result was also shown in a study that replaced magnetite sand with a cathode-ray tube up to 60% [106]. Although the 60% replacement resulted in a higher *μ* value, the compressive strength was reduced by 12.6%. This condition may be due to the smooth surface of the tube, which provides less traction and adhesion with the binder and other aggregates [177]. Furthermore, the tube has a lower mechanical strength than that of heavyweight concrete, and lead leaches from the tube, which adversely affected cement hydration and hence resulted in a lower compressive strength [65]. At a 80% steel shot replacement for limestone as the aggregate in concrete, a 38.1% reduction in compressive strength was recorded [120]. The lower mechanical strength of the steel shot was also shown by a study that replaced the steel shot with an electric arc furnace as the aggregate in the RSC [84]. The compressive strength exhibited a 22.5% increase with a 50% replacement of the steel shot with electric arc furnace slag. This result is due to the weaker strength of the steel shot compared with that of slag. Replacing the natural aggregate in concrete with copper slag also enhanced the compressive strength. The replacement of washed river sand with 60% copper slag resulted in a 13.9% gain in compressive strength [122]. This result is due to the angular form of copper slag particles, which enhanced the adhesion with the binder and other aggregates. This influence of particle angularity was also shown by a study that used a spherical form of copper slag, which resulted in the lower compressive strength of concrete than that of the natural aggregate concrete [95].

For RSC with added hydrogen, such as SBR and HDPE, no significant impact on the compressive strength of concrete was observed due to the presence of SBR or HDPE in the mixture. Increasing the SBR-to-cement percentage resulted in a varied 28-day compressive strength from 26–34 MPa [13]. This variation of compressive strength is also affected by the water-to-cement ratio, which also varied for individual samples. A sample with a 10% SBR-to-cement ratio and a water-to-cement ratio of 0.35 recorded the highest compressive strength at around 34 MPa, which is about the value of the sample with no SBR at a water-to-cement ratio of 0.4, thereby indicating a strength reduction due to the introduction of SBR into the composition. For concrete with HDPE replacing sand as the fine aggregate, the highest compressive strength was recorded for 30.9% by the volume of sand replacement in the concrete, which was 31.2 MPa [160]. This value is only 8% lower than that of the sample that contained natural aggregates only. 

Overall, the influence of the aggregate on the compressive strength of concrete is determined by its inertness, shape, surface condition, and strength [178]. The aggregate that is not inert and susceptible to chemical reactions, especially with cement, would affect the overall matrix of the hardened concrete. The shape and surface condition of the aggregate also affect the interlocking friction between the aggregates and the strength of the interfacial transition zone between the aggregate and cement paste. Lastly, the strength of the aggregate itself influences the resulting compressive strength by resisting the deformation of the overall concrete microstructure. Table 5 summarizes RSC with a compressive strength of less than 50 and above 50 MPa based on previous studies. The *μ* values listed in the table are based on exposure to 0.662 MeV of photon radiation. 

### 5.2. Splitting Tensile Strength

**Figure 15:**
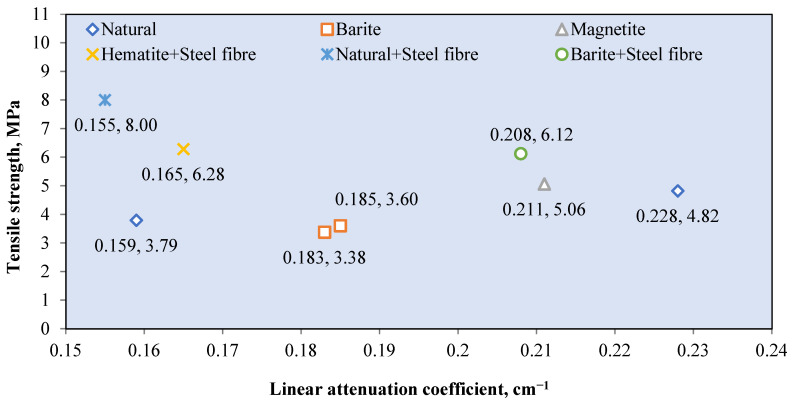
Tensile strength and linear attenuation coefficient of various RSCs (data adapted from [12,18,24,65,122,127]).

Figure 15 shows the performance of various RSCs on the basis of the linear attenuation coefficient and splitting tensile strength values. The attenuation coefficient is based on irradiation at a 0.662 MeV energy level. The highest splitting tensile strength is 8 MPa, whereas the lowest value is 2.6 MPa. Mostly, RSC attained a splitting tensile strength between 3–5 MPa (Table 6). A study on the effect of aggregate size on magnetite RSC recorded a maximum splitting tensile strength of 5.06 MPa with the maximum aggregate size of 12.5 mm [127].

The study indicates that an increase in the aggregate size resulted in the lower tensile strength of concrete due to increased porosity and a weakened interfacial transition zone. The use of micro-sized barite as the aggregate also resulted in a reduced splitting tensile strength. A study on barite powder replacing sand as the fine aggregate shows a 53% reduction in tensile strength due to a 50% replacement of sand with barite powder [75]. This condition is explained by the friability of barite and the weaker interfacial transition zone due to the powder form of barite. 

A splitting tensile strength of more than 5 MPa would contain at least 1% by volume of a steel fiber. A study on RSC with a natural aggregate and copper slag indicates that the addition of 1% by volume of steel fiber resulted in a 22.9% increment in the splitting tensile strength [122]. 

The study also noted that a 60% increase in the copper slag content resulted in a 25% increment in the splitting tensile strength, which indicates its superiority over natural sand. An increase in the percentage to 1.5% by volume of steel fiber resulted in a higher splitting tensile strength of 8 MPa for RSC with a natural aggregate [18]. On the basis of the same study, hematite and barite RSC recorded lower splitting tensile strengths of 6.28 and 6.12 MPa, respectively. Another study that used amang and lead glass reported a splitting tensile strength of 7 and 6.9 MPa, respectively, which are also lower than that of natural RSC with steel fiber [24]. 

### 5.3. Flexural Strength 

The effect of steel fiber on improving the tensile property of RSC is also reflected in the flexural performance. Table 7 shows the flexural value along with the photon shielding coefficient based on the 0.662 MeV energy level for RSC. The flexural strength of RSC reached up to 40 MPa with the incorporation of 143.3 kg/m^3^ or 2% by volume of steel fiber in the concrete [45]. The gain in the tensile and flexural properties of concrete that contains steel fiber is due to bridging on cracks provided by the steel fiber, which leads to restricted deformation and an overall higher tensile and flexural strength [179,180]. 

**Figure 16:**
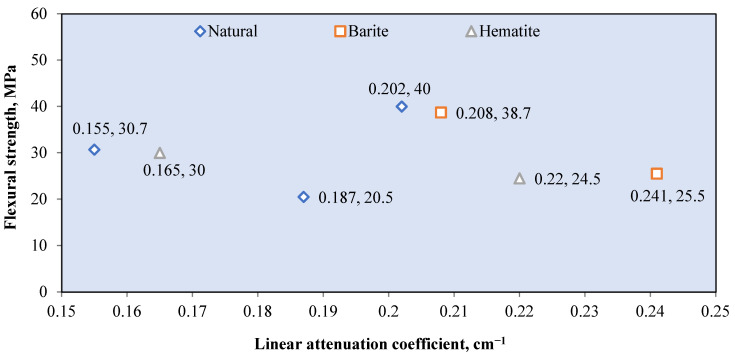
Flexural strength and linear attenuation coefficient based on 0.662 MeV gamma exposure on various RSCs with steel fiber additive (data adapted from [18,24,45,46]).

In terms of the type of aggregate used in RSC, previous studies showed that the replacement of the natural aggregate with waste or a heavyweight aggregate resulted in various effects on the flexural performance of the RSC. A study that used a 50% hematite replacement of dune sand resulted in a 4 MPa gain in flexural strength, while another study with 100% hematite as the aggregate reported a reduction of 0.7 MPa compared with silica sand RSC (Figure 16) [18,46]. For barite, a 100% replacement of quartz with barite as the aggregate in RSC resulted in an up to 36% reduction in flexural strength [45]. However, another study that produced barite RSC reported a flexural strength of 8 MPa, which is higher compared to that of silica sand concrete [18]. This result is due to the varied morphology and robustness of the heavyweight aggregate, which resulted in varied trends of changes in the flexural performance. One study that found an increase in the flexural strength due to hematite replacement is due to the significant gain in the fineness and angular morphology of the heavyweight aggregate [46]. This condition resulted in increased compactness and improved interlocking among compositions of the concrete matrix. In a study that observed a loss in flexural strength due to barite replacement, the drop in the flexural performance is due to the lack of robustness of barite compared with quartz, which contributed less in resisting flexural deformation [45]. 

The flexural strength of a waste aggregate such as amang and lead glass also shows a reduction in comparison with that of the natural aggregate of silica sand. Amang RSC and lead glass RSC recorded a 6.2% and 7.8% lower flexural strength compared with that of silica sand RSC, respectively [24]. On the basis of the study, the nature of lead glass is elongated and flattened, which may be the reducing factor on the flexural strength of RSC. In general, the mechanical properties of RSC are influenced by the material that constitutes the concrete matrix, which is also affected by the amount of water used for the binder. The superior mechanical properties of RSC will increase its durability against various possible threats to critical radioactive-related structures. Improved strength leads to improved security and confidence in the integrity of radiation shielding structures.

## 6. Durability Properties

The durability of RSC has been tested against elevated temperatures, freeze–thaw cycles, and chemical attacks. Other research also investigated the effect of aging on RSC, which is also an indicator of its durability. Studying the behavior of RSC at elevated temperatures provides indications of its durability. A primary containment concrete structure in a nuclear ionizing facility or the secondary containment of nuclear power generation is always exposed to elevated temperatures. A boiling water reactor typically operates at 287–304 °C; increasing the operating temperature would increase the thermal efficiency of the reactor in power generation [8]. Hence, this situation indicates the need for RSC with high thermal resistance. Investigating the durability of RSC against freeze–thaw cycles and chemical attacks helps measure its viability as exposed spent fuel storage, such as dry cask storage. 

### 6.1. Elevated Temperature

**Figure 17:**
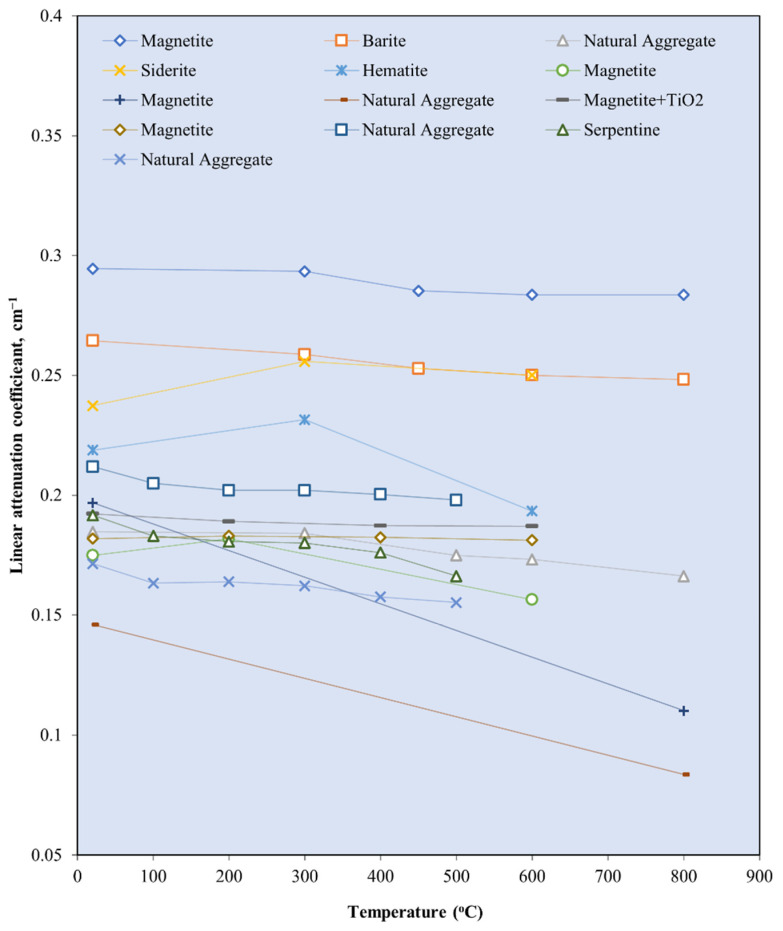
Relationship between linear attenuation coefficient (cm^−1^) based on 0.662 MeV gamma exposure and temperature (°C) of different types of concrete (data adapted from [16,44,53,67,123]).

Figure 17 shows the relationship between the *μ* value at 0.662 MeV of gamma ray exposure and temperature based on previous studies. In general, a temperature increase beyond 100 °C resulted in the reduced *μ* of concrete. Barite concrete recorded a 12.5% and 6.4% reduction in the *μ* value after being exposed to 450 °C and 800 °C, respectively [53,67] (Figure 10). In magnetite RSC, the reduction is minimal at 0.8% and 3.7% after exposure to 600 °C and 800 °C, respectively [16,53]. The larger reduction reported by barite RSC compared with magnetite RSC is due to the expansion of the barite aggregate, which leads to the cracking and spalling of the concrete sample [53,121]. 

A comparison of the coefficient of thermal expansion indicates that barite has almost three times the value of the natural aggregate and more than 1.5 times the value of magnetite [53,121,181]. The expansion of barite created cracks and resulted in a loss in the *μ* value [121]. For hematite RSC, exposure to 500 °C resulted in a 6.6% reduction in the *μ* value to 0.198 cm^−1^ [182]. This reduction is smaller than that of serpentine and dolomite RSC, which exhibited reductions of 9.4% and 13.1%, respectively, after exposure to 500 °C. Serpentine and dolomite concrete reported a residual *μ* value of 0.155 and 0.166 cm^−1^, respectively. The high reduction in the *μ* value of dolomite RSC could be attributed to the combustion of organic compounds inside it and water evaporation at a temperature range of 100 °C–600 °C [182]. For serpentine RSC, the reduction in the *μ* value is due to the loss of bonded water at a temperature of 500 °C because serpentine is a type of hydrated aggregate [119]. Goethite, which is another type of hydrated aggregate, also reported a loss of water, which led to a reduced *μ* value at an elevated temperature. 

Goethite RSC recorded a 15.6% reduction in the *μ* value after exposure to 450 °C, which is also a lower residual *μ* value than that of serpentine and dolomite concrete [67]. This condition is due to the de-hydroxylation and transformation of goethite, which is indicated by the largest weight loss of 11.25% after exposure to 450 °C [67]. The natural aggregate in RSC recorded a 10.3% loss in the *μ* value due to a crystalline transition, which led to a loss of density and, hence, a reduction in the attenuation property [53].

This condition is also shown in the large reduction in the *μ* value in the sample that contained a combination of fine-sized natural aggregate and coarse barite compared with barite RSC [53,67]. A study that used 6% nano-titanium as an additive to replace the cement of magnetite concrete reported a 2.5% reduction in the *μ* value compared with the 0.8% recorded by the control sample after exposure to 600 °C [16]. However, the study showed that an increase in the percentage of nano-titanium resulted in an increase in the residual *μ* value because of the non-porous structure of the concrete matrix contributed by the presence of nano-titanium and the ability of nano-titanium to withstand high temperatures.

Few studies reported an increment in the *μ* value with increasing temperature exposure. At 300 °C, the *μ* value of the RSC samples increased by 3.4–8.0%, with the heavyweight aggregate recording the largest increment compared to the natural aggregate [123]. This finding is also shown in the sample with the coarse barite and fine natural aggregate at 100 °C, which showed a 1.9% increase in the *μ* value. This condition may be due to the desiccation of the capillary water and the expansion of aggregates during the heating regime, which promotes compactness [123,183]. 

In contrast to normal-strength RSC, UHPRSC reported significant reductions in the *μ* value due to an elevated temperature. UHPRSC with a magnetite aggregate recorded a 44.2% reduction in the *μ* value, while UHPRSC with a natural aggregate reported a lower percentage of loss of 43.2% of the *μ* value [44]. These losses are reported at the exposure of 800 °C, where barite and magnetite RSC reported 6.4% and 3.7% lower *μ* values at the same temperature, respectively [53]. This large percentage of loss in the *μ* value as compared with that of lower-strength RSC is due to the dense packing of the UHPRSC matrix, which induced microcracks, hence reducing the attenuating ability [183,184]. Additional cracks due to the expansion of steel fiber and pores left by vaporized polyvinyl alcohol fiber in UHPRSC may also contribute to the significant reduction in the *μ* value of UHPRSC [185]. 

In term of neutron attenuation, a maximum loss of 30% in the ∑_R_ (E_n_) value is recorded by RSC, with the coarse barite aggregate exposed to 600 °C [123]. The lowest ∑_R_ (E_n_) reduction of 21% was recorded for the sample with the natural aggregate, followed by the sample with the siderite aggregate at 27.5% [123]. The study also reported large losses in neutron attenuation as compared with gamma ray attenuation of the sample due to elevated temperatures. This condition is due to depleted hydrogen in concrete as this element contributes largely in slowing down and absorbing free neutrons [123,186,187]. During heating, a high temperature desiccates pore water and results in the removal of chemically bound water from hydration products that contribute to the amount of hydrogen in the RSC composition [188,189]. The depletion of this element in RSC leads to reduced neutron attenuation. This reduction in the ∑_R_ (E_n_) value due to a loss of hydrogen is also reported in serpentine and goethite concrete. RSC with goethite as the coarse aggregate, which recorded the largest percentage of weight loss due to heat in the study, reported a 35.3% reduction in the ∑_R_ (E_n_) value after exposure to 450 °C [67]. It is the lowest reported residual ∑_R_ (E_n_) value in the study at 0.0717 cm^−1^, which is due to a loss of hydrogen nuclei in goethite from the evaporation of water and transformation to ferric oxide. Serpentine RSC, which recorded about a 15% weight loss at exposure to temperatures above 500 °C, indicated 0.07 cm^−1^ of a residual ∑_R_ (E_n_) value based on exposure to the same temperature [182]. This value is a 26% loss of the ∑_R_ (E_n_) value based on exposure to an Am–Be source.

High-temperature exposure reduces not only the shielding property of RSC, but also its mechanical properties. Exposure to 800 °C caused 79.6% and 84.8% losses in the compressive strength of barite RSC and natural aggregate RSC, respectively [53]. The residual compressive strength of barite RSC and natural aggregate RSC after heat exposure was 10 and 8 MPa, respectively. Magnetite RSC recorded the highest residual compressive strength after exposure to 800 °C, which was 11 MPa, but the percentage of loss compared with the original strength was 80.4%. The results of the residual compressive strength indicate that RSCs have a very low serviceability as structural members once exposed to elevated temperatures. To have sufficient residual compressive strength after heat exposure, concrete needs to have a higher initial compressive strength. This situation is shown by goethite and barite RSC, which have a low original strength of 38–42 MPa and ended up having a low residual compressive strength of 17–19 MPa after being exposed to only 400 °C [67]. Hence, RSC with an ultra-high strength such as UHPRSC would possess more practical residual strength after exposure to elevated temperatures. Research on UHPRSC reported that natural and magnetite aggregate UHPRSC have practical residual compressive strengths of 49 and 45 MPa, respectively [44]. These values were recorded after the samples were exposed to 800 °C despite a 66–68% loss of compressive strength. 

Even though UHPRSC has a higher residual compressive strength after heat treatment, the loss in the *μ* value is the highest among the reviewed studies. Hence, further research toward producing a higher residual compressive strength and radiation attenuation coefficient is required. RSC should be able to withstand external deteriorating elements and internal heat or combustion caused by any mishaps, while containing the radiation. Ultra-high-strength concrete such as UHPC outperforms normal-strength concrete under impact and blast, but suffers more spalling incidents at elevated temperatures, thus making it a suitable candidate as an RSC [190,191,192,193,194]. However, there is a dearth of research on UHPC’s radiation attenuation ability. Future research on improving its radiation shielding performance at higher temperatures may provide a superior alternative material for nuclear-related structures.

### 6.2. Freeze–Thaw Resistance

**Figure 18:**
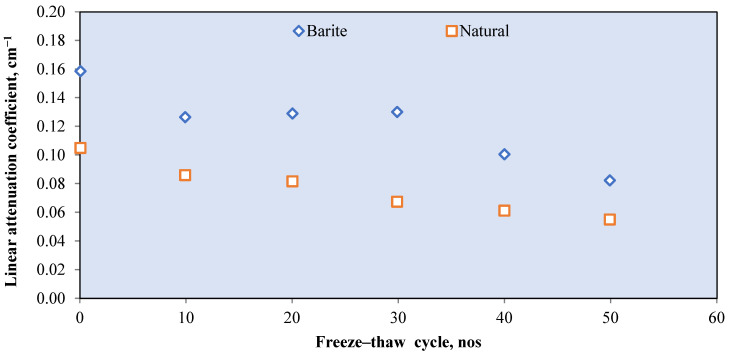
Value of linear attenuation coefficient based on exposure to freeze–thaw cycles (adapted with improvements from [195]).

Concrete is commonly used as the main material in the construction of buildings. Therefore, concrete production is important for critically vital buildings, such as hospitals and nuclear power stations. The freeze–thaw resistance of RSC is evaluated based on its residual linear attenuation coefficient after being exposed to freeze–thaw cycles. Figure 18 shows the results of using barite and natural aggregate RSC. The study indicates the 25–39% reduction in the *μ* value after 50 freeze–thaw cycles on barite RSC [195]. The *μ* value is based on exposure to a Co-60 source of 1.25 MeV of average energy on barite RSC, which is produced based on a water-to-cement ratio of 0.43–0.65. This result is almost similar to the natural aggregate concrete performance, which reported a 25–43% reduction in the *μ* value in the same range of water-to-cement ratio. On the basis of the study, the highest residual *μ* value recorded after 50 cycles by natural aggregate concrete was 0.07 cm^−1^, while barite concrete recorded 0.1 cm^−1^. This study found that a higher water-to-cement ratio could minimize the adverse effects of freeze–thaw cycles on the *μ* value. This finding is based on the lower reduction in the *μ* value in the sample with a higher water-to-cement ratio. A high water-to-cement ratio in concrete resulted in a high amount of free water in the capillary and porosity of concrete. The freezing of concrete caused water in the pores to freeze and expand. 

The expansion of water creates internal hydraulic pressure, which can be dissipated through the nearest voids. If this pressure exceeds the tensile strength of the paste, then cracks will appear from the point of failure [196]. Cracks increase and expand every freeze–thaw cycle, thereby reducing the shielding properties. A higher porosity due to a high water-to-cement ratio in the sample could dissipate the expanding water and reduce the internal pressure. This condition would minimize the formation of cracks and hence mitigate the reduction of shielding properties.

Moreover, barite is recognized as an excellent mineral for shielding γ-rays; however, with the difficulty of locating adequate barite deposits, barite is utilized only as an aggregate in concrete [197,198]. However, freeze–thaw cycles may damage the microstructure of concrete and thus affect the radiation shielding capability. The linear attenuation coefficient *µ* (cm^−1^), which is defined as the possibility of a radiation interaction with a material per unit path length, must be determined to evaluate the radiation shielding characteristics of building materials. The *µ* value is affected by the atomic number, incident photon energy, and shielding material density (ρ) [199]. A variety of theoretical and experimental studies on the linear attenuation coefficients of various types of concretes have been conducted. One study computed concrete’s linear attenuation coefficients with densities ranging from 10 keV to 1 GeV at a photon energy ranging from 10 keV to 1 GeV [105]. Another study estimated the linear attenuation coefficients for four different grades of concrete at the photon energy ranging from 10 keV–1 GeV [162]. The linear attenuation coefficients for various building materials have been determined recently, and the impact of barite on concrete with respect to radiation protection has been examined in other studies [162,197]. Although several studies on concrete have been conducted, including one on the impacts of freeze–thaw cycles on the mechanical characteristics of rocks [195,200,201], the lack of research on the effect of freeze–thaw cycles on radiation shielding characteristics prompted us to investigate the negative impacts of freeze–thaw cycles on concrete.

### 6.3. Chemical Resistance

**Figure 19:**
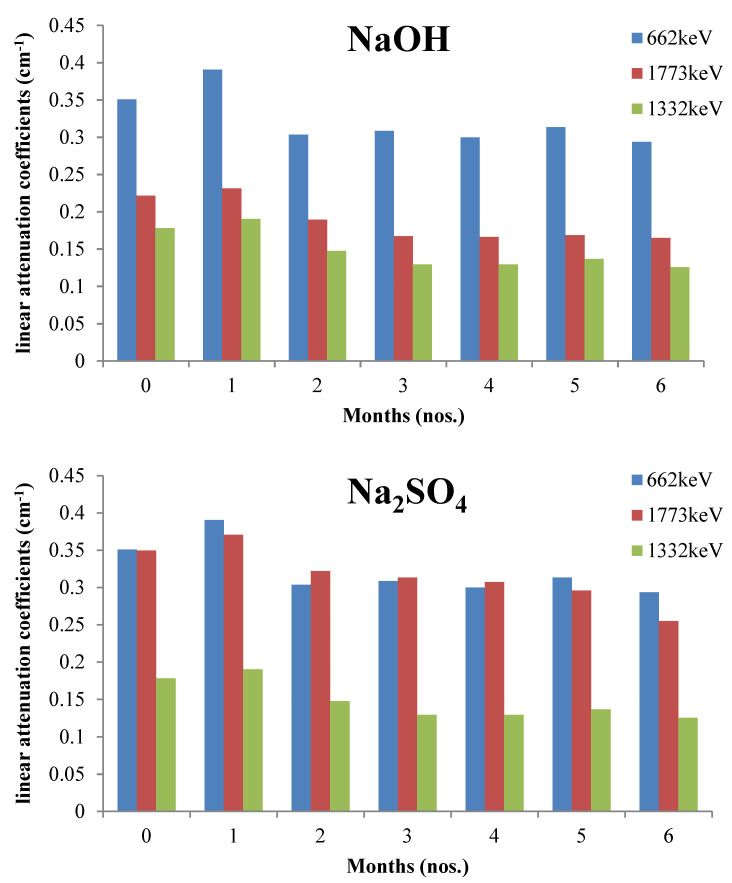
Variation of linear attenuation coefficients with distinct chemical environments at 662, 1332, and 1773 keV energies (adapted with improvement from [207]).

Radiation has been shown to have a deep influence on shielding materials beneath their chemical structure [202]. In metals, radiation displaces atoms from their equilibrium lattice locations, resulting in lattice defects, which are responsible for increased toughness, but also embrittlement, and thus, a loss of ductility [203,204]. The formation of extra cross-links in polymers is caused by the excess energy provided by radiation [205]. Nuclear radiation causes atomic molecules to break in geomaterials such as concrete, which is thought to underlie the degradation in the mechanical qualities of exposed concrete [206]. Reports on the chemical attack resistance of RSC are limited. A study on barite RSC reported a reduction in shielding properties due to immersion in sodium sulfate and sodium hydroxide [207]. The study shows that barite RSC had about 17% of its original *μ* value after being immersed in sodium hydroxide solution for 6 months. The *μ* value of 0.35 cm^−1^ is based on 662 keV of a Cs^−137^ source. Sodium sulfate also had a slightly greater adverse effect on the shielding properties of barite RSC, as indicated by the 28.6% reduction in the *μ* value to about 0.25 cm^−1^. The study concludes that intrusive chemical media adversely affect RSC. However, a detail that is worth noting is that the samples that were used in the study were produced with a water-to-cement ratio of 0.5, which is high and thus resulted in more porous concrete and a susceptibility to chemical attack with the variation of the linear attenuation coefficients with distinct chemical environments at 662, 1332, and 1773 keV energies (Figure 19) [207]. A sulfate attack on RSC caused expansion and higher permeability in RSC, resulting in higher expansion [208]. This expansion is caused by the formation of gypsum at the interfacial transition zone, which is the product of the reaction between external sulfate and calcium hydroxide in concrete [209]. This condition leads to the formation of cracks that originate from the interfacial transition zone, thereby reducing the shielding properties.

Another research that investigated the effect of chemical exposure to RSC indicates 20.5% and 59.5% reductions in the compressive and flexural strength of the RSC mortar, respectively, due to immersion in sodium sulfate for 90 days [210]. The study also shows that a 50% replacement of sand with eggshell resulted in 51.8% and 10.5% reductions in compressive and flexural strength, respectively. These larger reductions with the replacement of sand by eggshell mortar is due to the greater susceptibility of eggshells to a sulfate attack compared with sand, which is also indicated by the sizeable mass loss of 25.7% compared with 6.3% recorded by the sand mortar. However, a 50% replacement of sand with eggshells reported a higher *μ* value at 3.66 cm^−1^ compared with sand RSC at 1.49 cm^−1^. The *μ* value is measured based on low-energy Am-241 exposure at 26 keV. Overall, RSC would be susceptible to a chemical attack if it had a high porosity or contained materials that would react with the deteriorating chemical.

Experimental works on concrete under gamma irradiation allows investigators to conclude that interactions with the shielding material reduce both the porosity and strength of the material [211]. The mechanism is described as sequences of chemical reactions within the material, starting with the radiolysis of water and concluding with the formation of calcite (CaCO_3_). Calcite crystallites develop into a porous structure, reducing their size and destroying the tobermorite gel, a calcium silicate hydrate mineral liable for concrete strength, through crystallization pressure.

More recent research appears to support the radiolytic process [212], benefiting from both scanning electron microscopy and X-ray diffraction approaches. Gamma radiation absorption is discovered to produce the amorphization of cement hydrates and, eventually, their disintegration. Furthermore, bubbles were found following irradiation exposure, which is perhaps the result of chemically bound water separation, and various fractures in the cementitious matrix were also discovered. The transformation of crystalline quartz into deformed quartz has two negative consequences: (i) microcracking due to the variations of volume changes in the composite; and (ii) the increased reactivity to some aggressive chemicals, such as calcium hydroxide, which is responsible for alkali–silica reactions (ASR) in concrete [213]. Both of these impacts negatively affect irradiated concrete’s long-term performance. The stiffness of ASR gel is mostly determined by its chemical composition, including the Na_2_O/SiO_2_ ratio. The swelling pressure of ASR in relation to the ASR chemical composition has also been investigated [214]. Although this work did not directly address ASR, it showed that irradiation causes a series of chemical processes, leading to decreased pore space and thus preventing concrete from absorbing some of the ASR gel generated prior to expansion [211].

## 7. Applications of RSC

**Figure 20:**
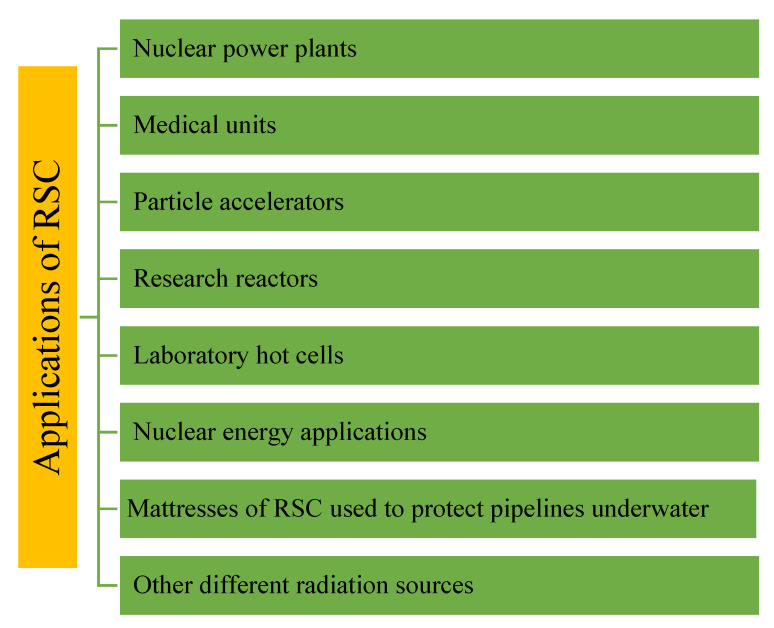
Applications of RSC.

RSC is a basic material for radiation protective shields because it has a good shielding ability, high durability, low cost, and high versatility, thus having the capacity to have structural functions that outperform the design standard [54]. RSC is generally applied to biological shielding in nuclear power plants, particle accelerators, medical units, laboratory hot cells, research reactors, and other types of radiation sources (Figure 20). For example, barite concrete is extensively used in Turkish hospitals and nuclear reactors [32]; in Britain, for the DIDO, BEPO, and PLUTO research reactors at Harwell [215,216,217]; and for making isotopes [80,182], where radioactive impenetrability is essential. This concept of shielding is also applied in shielding off electromagnetic waves (EMs) to protect sensitive electronic instruments and apparatus. Furthermore, concrete is also employed as shielding against EMs and the research shows promising results [218,219]. The shielding performance of concrete is improved with the incorporation of nano-material, as steel and glass provide higher EM shielding [220]. For RSC, it has been used as construction material for nuclear-related structures; more than 60% of light water reactor nuclear power plants in the United States are constructed using RSC [7]. RSC is employed in pressurized water reactor shield and boiling water reactor buildings. RSCs used in these nuclear power plant structures include reinforced concrete and post-tensioned concrete. Another example of a nuclear power plant built with RSC is the Pickering Nuclear Generating Station in Ontario, Canada, where RSC is used for containment structures, including reactor buildings, pressure relief ducts, and vacuum buildings [221]. RSC of a 1200 mm thickness is used to construct a perimeter wall that supports a 460 mm-thick RSC dome. RSC in these structures is required to have leak tightness, which is provided by the liner system composed of a 13 mm-thick steel plate or a carbon steel plate. 

RSC is also used in managing the spent nuclear fuel of nuclear power plants. Spent nuclear fuel is either treated as waste, which needs to be securely stored, or reprocessed to recover to any plutonium or uranium [10,222]. Spent nuclear fuel is stored in wet storage or dry storage structures made of RSC. Wet storage, which is also called a spent fuel pool, is constructed with RSC and lined with a steel plate to prevent leakages. This pool is filled with a liquid, such as water, which provides active cooling for heat-emitting spent fuels [8,60]. The emission of heat from spent fuel is due to the decaying process of the elements in the fuel. A dry storage system for spent nuclear fuel includes vaults, silo, and cask [60].

Spent fuel in dry cask storage is cooled passively by air convection. Dry cask storage is constructed with RSC, and it provides more mobility and radiation shielding than wet storage does. Dry cask storage will not generate liquid waste due to exposure to decaying spent fuel, thus making maintenance easy. Recent research on dry cask storage with RSC aimed at designing and measuring its durability against aging and untoward events [9,222].

In India, trenches for near-surface disposal facilities are made of RSC, with the wall thickness ranging from 350–750 mm [10]. This thickness depends on the depth of the trench, which is typically 4.8 m deep. This trench can be considered as dry storage; a typical trench requires adequate waterproofing to prevent the ingress of groundwater and mobile covers to protect against rain. A precast concrete slab is used as the top cover at the end of the filling operation. 

RSC is also used as a “hot cell”, which refers to an area that is designated to handle irradiated elements or waste [10,223]. Hot cells or high-activity handling cells use RSC as a shielding wall with an approximate thickness of 1.5 m. Activities in hot cells are monitored by using radiation shielding windows made of zinc bromide or by using a radiation-resistant camera. In this area, an irradiated fuel element and experimental rigs from nuclear facilities are dismantled remotely using a telerobot. RSC with an appropriate thickness provides shielding for the operator of the telerobot during the entire dismantling process.

In the health care sector, RSC is used to construct walls for radiotherapy rooms. A radiotherapy room is a facility that serves cancer patients and uses high-energy photons or protons [15]. A proton therapy center in Massachusetts General Hospital uses a 235 MeV cyclotron, while a radiotherapy facility at the Institute of Nuclear Physics in Krakow produces proton beams of 60 MeV [61,224]. In treating patients by using radiotherapy, high-energy photons penetrate the patient and some of the radiation is scattered, thus requiring shielding. The use of lead sheets in the room increased the neutron dose, and the use of steel sheets had an insignificant effect on the neutron dose [15]. Overall, RSCs are widely utilized in facilities that involve harmful radiation. It is a common source of shielding but requires more work in improving the shielding and mechanical performance without compromising its sustainability.

## 8. Conclusions

Based on previous work, RSC is a durable, functional, and cost-efficient material for radiation shielding infrastructures. It has been utilized for a variety of applications, including nuclear power plants, particle accelerators, research reactors, and high-level radioactive research facilities. However, there challenges regarding RSC that require future research; for example, it segregates easily as a result of the high density of the aggregates used. Improving the radiation shielding properties of RSC requires the incorporation of heavyweight material and substances that contain a neutron absorber. This replacement of a lightweight aggregate has an adverse impact on the mechanical strengths, especially compressive strength. 

The overall tensile and flexural strength is almost similar across many types of minerals, but the incorporation of steel fiber greatly improves these strengths. Exposure of RSC to a high temperature resulted in a minimal reduction of photon attenuation, but reduced the neutron attenuation coefficient significantly due to the desiccation of hydrogen. Mechanical strengths are also adversely affected by high temperatures, especially beyond 300 °C, because of the physical changes of the materials in the matrix of concrete. RSC experienced a reduction in the shielding efficiency and mechanical strengths due to the freeze–thaw cycles. Exposure to chemical attacks reduced the shielding properties of RSC due to internal expansion, which also negatively impacted the mechanical properties of RSC.

Recent research has focused on concrete’s ability to attenuate harmful energy that radiates from nuclear sources through various alterations to its composition. The last few decades have seen the development of RSC as a composite-based concrete with heavy natural aggregates such as magnetite or barites. RSC is deemed a superior alternative to many types of traditional normal concrete, having exceptional properties such as a high content of crystallized water and high density, and can thus be potentially applied to shield against various radiations such gamma rays, alpha rays, X-rays, and beta rays. In addition, RSC is less expensive, easier to mold into complicated shapes, and can be used for proton and neutron shielding. 

Given the merits of RSCs, this article presents a comprehensive review on the subject, considering the classifications, alternative materials, additives, and type of heavy aggregates used. This literature review also provides critical reviews on the RSC’s performance in terms of radiation shielding characteristics, strengths, and durability properties. Development research trends toward a broad understanding of the application possibilities of RSCs as advanced concrete products for producing a robust and green concrete composite for the construction of radiation shielding facilities are also extensively reviewed. Furthermore, this critical review provides a view of the progress made on RSCs and proposes avenues for future research with respect to this hotspot topic, including improving the durability of UHPRSC, which is imperative to producing superior and sustainable RSC. 

Moreover, it is also found that RSC decreases the intensity of radiation, relying on the density and thickness of the concrete structure, exhibiting that a higher density material can lower the radiation faster. In conclusion, this comprehensive review study was mainly written for engineers, and deals with the development and construction of concrete shielding; however, the manuscript also summarizes widely-dispersed data and information accessible on this subject, as an inclusive insight that must, therefore, be beneficial to scholars and to those interested in conducting value-added research in this hotspot research topic.

On the basis of this comprehensive review, RSC requires more work to further improve its performance. This includes its mechanical, radiation shielding and sustainability. Current research recorded a lower mechanical strength compared to advance concrete such as UHPFRC. Hence, several hotspot research topics are inspired and recommended for further consideration in future studies and investigations by researchers worldwide:

− UHPRSC using artificial and sustainable aggregate needs to be developed to broaden its application, especially in efficient nuclear power generation.
− More research is needed to determine the influence of binding materials, particularly active mineral additives, on shielding characteristics.
− Research on the influence of freeze–thaw cycles on radiation shielding properties is lacking. Therefore, further study on the detrimental effects of freeze–thaw cycles on RSC is highly needed.
− The usage of a shielding-improving additive such as nano-TiO_2_ in UHPRSC to further improve its shielding without compromising the high mechanical performance needs to be investigated.
− Research is lacking on the durability and any possibility of the crack-sealing property in UHPRSC, which provides an advantage to resist aging and extend its service period as a shielding structure.
− Development of sustainable RSC, such as geopolymer RSC, with various types of additives and aggregates to optimize its shielding and mechanical performance is highly needed.
− RSC has a higher demand for a pile foundation and has a negative influence on earthquake resistance due to its large dead weight.
− Development of an aging simulation on RSC that can precisely measure its reaction due to a long aging period, which is beneficial in mitigating structure failure and providing a rehabilitation program for aged structures, is highly imperative.
− An urgent investigation on sustainable heavyweight and neutron-absorbing additives, especially from processed waste or refined industrial by-products, is required.
− The long-term characteristics of RSC after exposure to various rays are critical to safety and must be thoroughly explored.

## Figures and Tables

**Table 1:** Composition, performance, and density of heavyweight aggregates.

Types of Aggregates	Relative Density	Chemical Composition of Principal Mineral	Performance	Refs.
Magnetite	4.6–5.2	Fe_3_O_4_	Shield gamma rays	[68]
Barite	4.0–4.4	BaSO_4_		
Hematite	4.6–5.2	Fe_2_O_3_		[69]
Serpentine	2.4–2.65	Mg_3_Si_2_O_5_(OH)_4_		
Ilmenite	4.2–4.8	FeTiO_3_	Shield neurons	[70]
Ascharite	3.4–3.6	Mg_2_B_2_O_5_·H_2_O		
Limonite	3.4–4.0	Hydrous iron ores containing 8–12% water		[71]
Lepidocrocite	-			
Basalt	2.6–2.8	–	Shield X-rays	[11]

**Table 2:** Summary of radiation test setup.

Radiation Source	Distance from Source to Specimen (mm)	Distance from Detector to Specimen (mm)	Thickness of Sample, mm	Detector Type, mm	Dia. of Collimator Opening, mm	Duration of Count Observation, min	Ref.
Cs137, Co60	20	50	150	50.8 × 50.8 NaI(Tl)	-	-	[95]
Cs137, Co60	-	-	-	76.2 × 76.2 NaI (TL)	18	-	[111]
Cs137, Co60	-	-	20–100	101.2 dia. NaI(Tl)		15 min	[80]
Cs137	330	310	11–16	-	2.8	-	[112]
Cs137	500	500	-	Berthold LB-6411	-	-	[103,113,114]
Cs137, Co60	147	286		50.8 × 50.8 NaI(Tl)	26	1.5 min	[62]
Cs137, Co60	-	-	20–100	76.2 × 76.2 NaI(Tl)			[38]
Cs137	100	100	10–90	50.8 × 50.8 NaI(Tl)	5		[115]
Co60	100	50	12–36	HPGe detector			[23]
Co60	790	60	26–182 mm				[91]
Ba133, Am241, Co60	100	50		NaI(Tl)	Pin hole, (1 cm^2^)		[108,116,117]
Cs137, Co60	20	50	10–40	76.2 × 76.2 NaI(Tl)	10 slit	-	[20]
Cs137	-	350	80	Scintillation type detector	-	60 min	[53]
Co60	200	200	20	HPGe type detector	-	-	[81]
Cs137, Co60,	50	400	40–120	Scintillator 40 × 40	-	120 min	[109]

**Table 3:** List of samples with major comprising elements from reviewed papers.

Sample Name	Aggregate	Fe_2_O_3_ (%)	Fe_3_SO_4_ (%)	BaSO_4_/BaO (%)	Linear Attenuation Coefficient, *μ* (cm^−1^)	Aggregate Volume by Weight in Mix (kg/m^3^)	Ref.
B-UHPC	Barite	-	-	58.69	0.208	1630	[18]
BL	Barite	-	-	20.84	0.0918	1798.3	[67]
B	Barite	-	-	74.31	0.241	1444	[45]
BC	Barite	-	-	90.3	0.265	2920	[53]
MC	Magnetite	-	90.8	-	0.295	3320	[53]
S1	Magnetite	72.1	-	-	0.228	3378.3	[12]
H-UHPC	Hematite	71.71	-	-	0.165	1100	[18]
G.L	Geothite	67.0	-	-	0.0822	1651.3	[67]

**Table 4:** Macroscopic removal cross section value concrete sample with different types of aggregate.

Sample	Source	Energy (MeV)	∑_R_ (E_n_) (cm^−1^)	Aggregate	Mass of Aggregate per Cubic Meter of Concrete (kg/m^3^)	Refs.
CC1	Am–Be	4	0.075	Natural	1762.7	[13]
CC	Am–Be	4	0.104	Granite	1363.9	[160]
NC	Am–Be	0.025	0.133	Natural	1746	[123]
PC00	Am–Be		0.13954	Limestone	1812.5	[81]
BC	Am–Be	0.025	0.15	Barite	2673	[123]
B100	Am–Be	4.5		Barite		[161]
M	Pu–Be		0.0996	Magnetite	2078	[163]
B85C15	Am–Be	4.5		Barite (85–95%) + colemanite (5–10%)		[161]
N85C15	Am–Be	4.5		Natural (85–95%) + colemanite (5–15%)		[161]
FCL	Am–Be	4.5	0.148	Limonite	2369.28	[165]
HC50	Am–Be		0.14112	Limestone + hematite	1254.7	[81]
Peridotite Concrete	Am–Be		0.1445	Peridotite	1703	[166]
A	Pu–Be	4	0.0922	Serpentine	1556.1	[110]
ABC	Pu–Be	0.8–11	0.0702–0.0922	Serpentine	1856.1	[109]
AB 25, AB50	Pu–Be	4	0.1226–0.1484	Serpentine +barite (25–50%)	1538.61	[110]
AH25, AH50	Pu–Be	4	0.1105–0.1398	Serpentine + hematite (25–50%)	1429.05	[110]
SC	Am–Be	0.025	0.1525	Siderite + barite	2520	[123]
L1	Am–Be	4	0.069–0.09	Natural aggregate + SBR	1765.2	[13]
MP1-MP9	Am–Be	4	0.1077–0.1103	Granite + HDPE	1274.7	[160]
CFM	reactor ET-RR-1	2–15	0.196	SBR + magnetite + boron carbide		[64]
MPCC1G1.0	Pu–Be		0.1034	Magnetite + acrylic + gadolinium	2078	[163]

**Table 5:** Compressive strength of RSC.

RSC with Compressive Strength of Less than 50 MPa						
Sample	Density (kg/m^3^)		Compressive Strength (MPa)	*μ* (cm^−1^)	Aggregate	Ref.
50B50LS	3270		33.1	0.211	Barite	[88]
100B	3610		32.9	0.236	Barite	[88]
BC	3441		49	0.265	Barite	[53]
CB-FA-50	3230		32	0.175	Barite	[65]
CB-0	3410		35	0.185	Barite	[65]
B.L	2963		38	0.092	Barite	[67]
G.L	2906		41	0.082	Geothite	[67]
IO100	3029		40	0.160	Iron ore	[120]
BW6	3740		44.1	0.198	Magnetite + Bi_2_O_3_ (3%) + WO3 (3%)	[14]
M-4	2620		34.1	0.194	Magnetite	[106]
O	3708		40.7	0.186	Magnetite	[14]
100MW	3040		43.84	0.196	Mine waste	[88]
LSA100	2435		42	0.120	Natural	[120]
100LS	2990		37.7	0.184	Natural	[88]
s4	3687		41	0.221	Natural	[12]
G0C0	2296		35.7	0.245	Natural	[95]
G60C100	2845		32.4	0.333	Slag	[95]
7			46.5	0.201	Slag	[128]
S.L	2994		49	0.083	Slag	[67]
SA100	2790		45	0.140	Slag	[120]
SS100	3563		30	0.200	Steel shot	[120]
RSC with compressive strength of greater than 50 MPa						
Sample	Density (kg/m^3^)	w/c	Compressive strength (Mpa)	*μ* (0.662 Mev)	Aggregate	Ref.
BC	3311.85	0.4	58.1	0.234	Barite	[123]
B-UHPC	3112	0.16	138	0.208	Barite	[18]
B	2943	0.18	172	0.241	Barite	[45]
Q+B	2684	0.18	190	0.221	Barite	[45]
D.C	2570	0.43	51.0	0.0797	Dolomite	[67]
H-UHPC	2602	0.19	149	0.165	Hematite	[18]
HP-50	2900	0.17	170	0.022	Hematite + Silica	[46]
SC	3158.85	0.4	63.8	0.22	Siderite	[123]
M-U		0.16	134	0.197	Magnetite	[44]
MC	3939	0.35	56.0	0.295	Magnetite	[53]
70NCA	2398	0.23	87.0	0.1849	Natural	[66]
s1	3871	0.4	62.0	0.228	Natural	[12]
M-0.35/CS0	2542	0.35	67.4	0.15914	Natural	[122]
SS-UHPC	2401	0.16	165.7	0.155	Natural	[24]
Q	2438	0.18	218	0.202	Natural	[45]
SS-UHPC	2401	0.16	166	0.155	Natural	[18]
HP-0	2500	0.14	160	0.0187	Natural	[46]
70RCA	2321	0.23	80	0.1743	Recycled aggregate	[66]
40RCA	2289	0.37	50	0.1723	Recycled aggregate	[66]
M-0.35/CS60/SF	2668	0.35	78.62	0.23771	Slag	[122]
LG-UHPC	2479	0.17	170.1	0.175	Lead glass	[24]
A-UHPC	3036	0.16	157.5	0.182	Mine waste	[24]

**Table 6:** List of RSC with tensile strength value.

Name	Steel Fiber (%)	LAC (cm^−1^)	Aggregate	Tensile Strength (MPa)	Ref.
S1	0	0.228	Natural	4.82	[12]
M-0.35/CS0	0	0.159	Natural	3.79	[122]
CB-0	0	0.185	Barite	3.6	[65]
CB-CA-10	0	0.183	Barite + CRT 10%	3.375	[65]
D12.5W0.45C400	0	0.211	Magnetite	5.06	[127]
G30C0	0	0.254	Natural + GGBS	4.93	[95]
G0C100	0	0.32	Copper slag	4.18	[95]
G30C100	0	0.328	Copper slag + GGBS	3.9	[95]
M-0.35/CS60/SF	1	0.2377	Natural + copper slag (60%)	5.79	[122]
M-0.40/CS60/SF	1	0.2296	Natural + copper slag (60%)	5.46	[122]
A-UHPC	1.5	0.182	Amang	7	[24]
LG-UHPC	1.5	0.175	Lead glass	6.9	[24]
H-UHPC	1.5	0.165	Hematite	6.28	[18]
B-UHPC	1.5	0.208	Barite	6.12	[18]
SS-UHPC	1.5	0.155	Silica sand	8	[24]

**Table 7:** List of RSC with flexural strength value.

Name	Steel Fiber (%)	LAC (cm^−1^)	Aggregate	Flexural Strength (MPa)	Ref.
M-0.35/CS60/SF	1	0.2377	Natural + copper slag (60%)	8.27	[122]
M-0.40/CS60/SF	1	0.2296	Natural + copper slag (60%)	7.65	[122]
A-UHPC	1.5	0.182	Amang	28.8	[24]
LG-UHPC	1.5	0.175	Lead glass	28.3	[24]
H-UHPC	1.5	0.165	Hematite	30	[18]
B-UHPC	1.5	0.208	Barite	38.7	[18]
SS-UHPC	1.5	0.155	Silica sand	30.7	[24]
Q	2	0.202	Quartz	40	[45]
B	2	0.241	Barite	25.5	[45]
HP-0	1.9	0.187	Dune sand	20.5	[46]
HP-50	1.9	0.22	Natural + Hematite (50%)	24.5	[46]

## Data Availability

Data sharing not applicable.
